# Dynamic Remodeling of Oocyte‐Granulosa Cell Communication During Bovine Folliculogenesis Revealed by Transcriptomic Meta‐Analyses

**DOI:** 10.1096/fj.202603316RR

**Published:** 2026-09-11

**Authors:** Noemi Monferini, Ludovica Donadini, Pritha Dey, Federica Franciosi, Valentina Lodde, Maria Belen Rabaglino, Alberto Maria Luciano

**Affiliations:** ^1^ Reproductive and Developmental Biology Laboratory (ReDBioLab), Department of Veterinary Medicine and Animal Sciences University of Milan Milan Italy; ^2^ Department of Population Health Sciences, Faculty of Veterinary Medicine Utrecht University Utrecht the Netherlands

**Keywords:** cell–cell signaling ligand–receptor interactions, cumulus cell, developmental competence, folliculogenesis, oocyte–granulosa cell communication, ovary

## Abstract

Ovarian folliculogenesis relies on tightly coordinated communication between the oocyte and surrounding granulosa cells, yet how this molecular dialogue is remodeled during follicle development remains poorly understood. Here, we reconstructed stage‐specific ligand–receptor communication networks through a transcriptomic meta‐analysis integrating bovine secondary, early antral, and middle antral follicles. Our analyses revealed that oocyte–granulosa cell communication undergoes progressive remodeling during folliculogenesis, with distinct signaling programs characterizing successive developmental stages. Secondary follicles were predominantly associated with extracellular matrix organization, cell adhesion, and early metabolic regulation. During the early antral stage, signaling shifted toward lipid, steroid, and vitamin metabolism, identifying this phase as a major metabolic transition. Middle antral follicles exhibited a marked increase in communication complexity, with enrichment of PI3K–AKT, mTOR, RAS, Hippo, and cell adhesion pathways accompanying the acquisition of developmental competence. Additional analyses of Brilliant Cresyl Blue‐classified cumulus–oocyte complexes identified competence‐associated ligand‐receptor interactions, while independent validation using the EmbryoGENE dataset confirmed stage‐specific expression patterns and highlighted *CD47*, *FGF21*, and *GPC6* as candidate regulators of oocyte developmental competence. This study provides a comprehensive transcriptomic framework describing the dynamic remodeling of oocyte–granulosa cell communication during bovine folliculogenesis. Beyond confirming established signaling pathways, it identifies novel candidate interactions and offers a biologically grounded resource to guide future functional studies and the optimization of in vitro follicle and cumulus–oocyte complex culture systems.

## Graphical abstract

Bidirectional communication between oocytes and granulosa cells undergoes progressive remodeling during bovine folliculogenesis. Integrative transcriptomic analysis identified stage‐specific ligand‐receptor signaling networks coordinating structural, metabolic, and developmental processes. Independent validation using oocyte chromatin configuration confirmed candidate regulators associated with oocyte developmental competence.

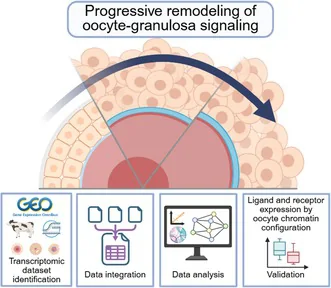

## 1 Introduction

The ovary is a highly dynamic organ whose plasticity is largely driven by the coexistence of oocytes at different developmental stages [1]. Each oocyte is embedded within and differentially supported by surrounding granulosa cells, together forming the ovarian follicle. Most follicles reside in the preantral stage and progressively develop by forming the antral cavity, differentiating granulosa cells into cumulus granulosa cells (cGC) and mural granulosa cells (mGC), acquiring increasing metabolic competence, and becoming dependent on gonadotropins [2, 3].

For many years, research has focused primarily on antral follicles, largely because of their relevance to assisted reproductive technologies. However, attention is increasingly shifting toward preantral follicles, the most abundant follicular population, which thus constitute a potentially valuable source of oocytes [4, 5, 6]. Despite this interest, only a limited number of approaches are currently available to advance preantral follicles to the antral stage, owing to an incomplete understanding of the molecular mechanisms governing the early phases of folliculogenesis [7, 8].

In previous studies, we identified several mechanisms involved in bovine preantral follicle growth, spanning from the primordial stage through secondary follicles (SF) to the antral stage [9, 10]. Nevertheless, the molecular interactions that specifically regulate the transition from the preantral to the antral stage in bovine follicles remain largely unexplored.

Preantral follicle development requires tightly regulated gene expression at each follicular stage, establishing finely orchestrated communication between the germinal and somatic compartments [11]. Bidirectional interactions between the oocyte and granulosa cells are essential for follicular growth and are mediated by autocrine and paracrine signaling via secreted factors, as well as by direct juxtacrine communication mechanisms such as gap junctions [1, 11, 12, 13, 14, 15].

Several key signaling axes, including TGFβ, KIT, and cyclic nucleotides, have been identified as important mediators of oocyte‐granulosa cell crosstalk [1, 16, 17, 18, 19]. However, many additional mechanisms remain unknown, particularly during the earliest stages of folliculogenesis. Communication between the two compartments is initiated as early as the preantral stage and progressively evolves and diversifies throughout follicular development [20, 21]. Several studies have investigated the molecular signatures of the germinal compartment, the oocytes [22], and the somatic compartment, comprising granulosa cells and cGC [23, 24, 25], outlining the key transcriptomic changes occurring in these cellular compartments during follicular development. To date, however, a comprehensive understanding of the germinal‐somatic dialogue during folliculogenesis is still lacking.

In this study, we aimed to characterize oocyte–granulosa cell interactions from a transcriptomic perspective by identifying ligands and receptors expressed in secondary (SF), early antral (EA), and middle antral (MA) follicles using a unified bioinformatic framework. To strengthen the robustness of the inferred communication networks, we integrated our own transcriptomic data with publicly available datasets representing key developmental transitions throughout folliculogenesis. This approach enhances the detection of reproducible biological signals while minimizing study‐specific technical variation, thereby providing a comprehensive view of the molecular dialogue between the oocyte and its surrounding granulosa cells.

Building upon previous transcriptomic studies of individual follicular stages and cellular compartments, we hypothesized that ligand‐receptor communication is progressively remodeled as follicles acquire structural organization, metabolic competence, and developmental potential. Accordingly, the present study was designed as a transcriptomic discovery framework to reconstruct stage‐specific communication networks and prioritize biologically plausible ligand‐receptor interactions for subsequent functional investigation. Elucidating this molecular dialogue is essential for understanding the mechanisms coordinating folliculogenesis and provides a rational basis for improving in vitro follicle and cumulus‐oocyte complex culture systems.

## 2 Materials and Methods

**FIGURE 1:**
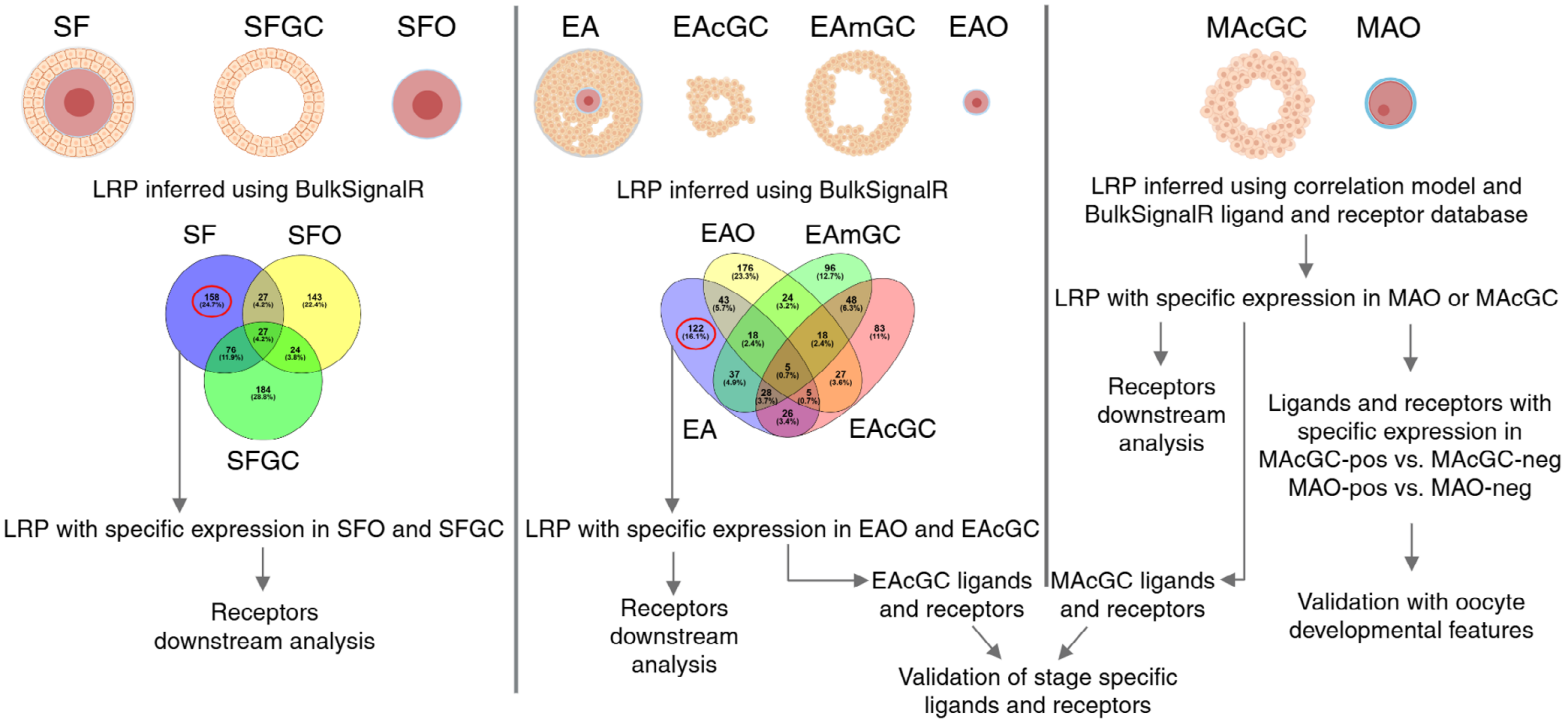
Data analysis pipeline. The scheme illustrates the workflow used to analyze ligand‐receptor pair (LRP) interactions in bovine follicles from secondary follicles (SF) to early (EA) and middle antral follicles (MA) stage. Ligand and receptor information was retrieved from the BulkSignalR package, and a correlation test was applied to identify putative LRP. Venn diagrams depict the subtraction of LRP determined within samples of oocyte (secondary follicle oocyte, SFO; early antral follicle oocyte, EAO) and granulosa cells (secondary follicle granulosa cells, SFGC; early antral follicle cumulus granulosa cells, EAcGC; early antral follicle mural granulosa cells, EAmGC) from the LRP obtained within follicle samples (SF and EA). In MA stage, a correlation analysis of the ligand and receptor identified using the BulkSignalR database between middle antral follicle oocyte (MAO) and middle antral follicle cumulus granulosa cells (MAcGC) was conducted. Downstream and validation analyses were subsequently performed on the identified LRP. Created in BioRender. Monferini, N. (2026) https://BioRender.com/8vyaj8y.

The data of follicles and follicle compartments used in this study were downloaded from the public functional genomic data repository Gene Expression Omnibus (GEO) [26]: GSE255879 [25], GSE249434 [22], GSE199210 [23], in addition to our own data (GSE309360 and GSE309598) [10]. More detailed characteristics about each study are presented in Table 1, together with the description for each abbreviation used throughout the text. Integration of follicle datasets at different developmental stages was implemented using the R programming language (v4.5.1) [27] and R packages. A pipeline of the analyses performed is represented in Figure 1.

**TABLE 1:** Overview of the datasets employed for data analysis.

References	GEO accession number	Sample group	Description	*N*
Monferini N, Dey P et al., 2026 [10]	GSE309360	SF	Mechanically isolated secondary follicles	3
Donadini L et al., − *requested*	GSE309598	SF	Mechanically isolated secondary follicles	4
		EA	Cumulus granulosa cells‐oocyte complex and mural granulosa cells isolated from early antral follicles	4
Fushii M et al., 2024 [25]	GSE255879	SFGC	Granulosa cells from secondary follicles	4
		EAcGC	Cumulus granulosa cells from early antral follicles	5
		EAmGC	Mural granulosa cells from early antral follicles	5
Barbosa Latorraca L et al. 2024 [22]	GSE249434	SFO	Oocyte (< 60 μm) from secondary follicle	21
		EAO	Oocyte (100–109 μm) from early antral follicle	27
Walker BN et al., 2022 [23]	GSE199210	MAO‐neg	BCB‐ oocyte from middle antral follicles COCs	10
		MAcGC‐neg	Cumulus granulosa cells surrounding BCB‐ oocyte from middle antral follicles COCs	10
		MAO‐pos	BCB+ oocyte from middle antral follicles COCs	9
		MAcGC‐pos	Cumulus granulosa cells surrounding BCB+ oocyte from middle antral follicles COCs	9

### 2.1 Bulk Inference of Ligand‐Receptor Interactions in SF and EA Using BulkSignalR

For the SF category, since the datasets were from two different studies, the batch effect was first corrected using ComBat‐seq from the sva package (v3.52.0) [28]. All count data (SF, SFO, SFGC, EA, EAO, EAcGC, and EAmGC) were transformed using the variance‐stabilizing transformation (VST) of DESeq2 package (v1.44.0) [29]. The category from each experimental group were analyzed separately for the following steps.

The BulkSignalR package (v1.0.4) [30] was employed to identify and characterize ligand‐receptor pair (LRP) interactions within each category. This approach relies on bulk RNA‐seq data and quantifies correlations among ligand and receptor expression and the expression of receptors' target genes in downstream pathways. BulkSignalR was selected because it jointly considers ligand expression, receptor expression, and downstream target gene activity, enabling inference of biologically coherent intercellular signaling networks from bulk transcriptomic datasets. LRPs identified at each stage between the oocyte and granulosa cells were subsequently compared using Venn diagrams in Venny (v2.1.0) [31] to determine overlapped pairs across stages. To exclude LRPs expressed exclusively by one follicular compartment, we retained only those LRPs identified in SF that were absent from both the SFO and SFGC datasets. In other words, among the LRPs detected in SF, SFO, and SFGC, we selected only those uniquely identified in SF, as these are more likely to represent signaling interactions occurring between the oocyte and granulosa cells rather than expression originating from either cell type alone. Similarly, among the LRPs detected in EA, EAO, EAcGC, and EAmGC, we retained only those uniquely identified in EA, as candidate LRPs potentially reflecting communication between the oocyte and cumulus/mural granulosa cells. Next, to further corroborate the LRPs involved in the interaction between somatic and germinal cells, we analyzed the expression patterns of the filtered SF and EA ligands and receptors. Differential expression analyses were performed between expression SFGC vs. SFO and EAcGC vs. EAO using an ANOVA test, after normalizing raw counts with the log_2_‐transformed counts per million (log_2_CPM) method implemented in the edgeR package (v4.6.3) [32] (Figure 1). Genes showing significant differential expression (Benjamini–Hochberg adjusted *p* < 0.05) were then matched to the LRP identified in BulkSignalR. This significance threshold was adopted to control the false discovery rate while retaining biologically meaningful candidate interactions. Only LRPs in which both genes were significantly differentially expressed—with one gene predominantly expressed in oocyte and the other in granulosa cells—were retained as candidate germinal‐somatic cell interactions. The resulting ligand‐receptor interactions were visualized in circle plots using circlize (v0.4.16) [33].

### 2.2 Inference of Ligand‐Receptor Interactions in COCs Derived From MA Follicles (MA COCs)

Unlike the SF and EA stages, no bulk RNA‐seq datasets from intact bovine middle antral (MA) follicles are currently available in public repositories. Consequently, ligand‐receptor interactions at this stage could not be inferred using the BulkSignalR workflow applied to whole follicles. Instead, we analyzed paired RNA‐seq datasets from middle antral oocytes (MAO) and their corresponding cumulus granulosa cells (MAcGC) isolated from the same cumulus‐oocyte complexes (COCs). Candidate ligand‐receptor interactions were inferred by combining the BulkSignalR ligand‐receptor database (LRdb) with gene expression correlation analysis. The COCs had previously been classified by Brilliant Cresyl Blue (BCB) staining, which distinguishes fully grown, developmentally competent oocytes from growing oocytes based on glucose‐6‐phosphate dehydrogenase (G6PDH) activity [34]. Fully grown oocytes have a low G6PDH activity, so they do not degrade the BCB and therefore retain the blue/purple coloration (BCB‐positive, BCB‐pos), whereas growing oocytes display high G6PDH activity, which reduces the dye and results in the loss of staining (BCB‐negative, BCB‐neg). Moreover, BCB‐positive oocytes have a different pattern of co‐expressed genes compared with BCB‐negative [23], resulting in a higher maturation and blastocyst rate [34, 35, 36] and cloning efficiency [37].

To this end, gene expression from each dataset was normalized using ComBat‐seq, treating cumulus cells and oocytes originating from the same COC as a shared batch, while specifying cell type as the biological variable of interest in the model. The normalized counts were then log_2_ transformed. The ligand‐receptor database (*LRdb*) implemented by BulkSignalR was employed to obtain the reference set of LRPs. Next, the MAO and MAcGC matrices were filtered to retain the genes involved in the LRPs. Finally, Pearson correlation between MAcGC and MAO expression was computed using the WGCNA package (v1.73) [38]. Only positive correlations with a *p* < 0.05 were retained as our objective was to identify candidate ligand‐receptor interactions showing coordinated expression between the germinal and somatic compartments. Additionally, a one‐way ANOVA was performed to compare expression levels between MAcGC and MAO. Correlations were further filtered to retain only those LRPs involving genes showing significant differences between MAcGC and MAO (adjusted *p*‐value < 0.05). Ligand‐receptor interactions were visualized as circle plots generated with circlize package [33], whereas correlation analysis results were plotted using ggplot2 (v4.0.0).

Furthermore, the expression levels of genes involved in significant LRPs were compared using one‐way ANOVA across BCB staining groups (MAcGC‐neg vs. MAcGC‐pos and MAO‐neg vs. MAO‐pos) to assess whether ligands or receptors exhibited BCB‐specific expression patterns. Differences were considered significant at an adjusted *p*‐value < 0.05.

### 2.3 Downstream Analysis of Receptor‐Mediated Signaling

To investigate the biological processes potentially associated with the identified LRP, receptor genes were analyzed using the GeneMANIA plugin [39] implemented in Cytoscape v3.9.1 [40]. GeneMANIA integrates evidence from physical interactions, genetic interactions, pathway co‐membership, and computational predictions to identify genes functionally associated with the receptors of interest.

For each receptor, interaction networks were generated using the 30 highest‐ranked associated genes, a number selected to provide an interpretable network while retaining the principal receptor‐associated interactions. Functional enrichment analysis was then performed, and the 20 highest‐ranked functional attributes were retained to summarize the predominant biological processes represented within each network. Automatic weighting was applied, and *Homo sapiens* was selected as the reference organism because human annotations currently provide substantially more comprehensive interaction and pathway information than the corresponding bovine databases.

### 2.4 Cumulus Granulosa Cell Ligand and Receptor Validation With EmbryoGENE Dataset

Because of the intimate bidirectional interaction between somatic and germinal compartments, molecular features of cumulus granulosa cells have been proposed as indirect indicators of oocyte quality and functional status. Here, we investigated whether the patterns of ligand and receptor expression in cumulus granulosa cells reflect the chromatin state of the oocyte they surround. To do this, we compared these expression patterns with data from the EmbryoGENE dataset (https://emb‐bioinfo.fsaa.ulaval.ca/IMAGE/index.html); only probes annotated as constitutive were included [41], in which both oocytes and cumulus granulosa cells are classified according to the oocyte's chromatin configuration [34, 41].

First, to assess whether the identified ligands and receptors were specific to the EA and MA stages, we compared our results with microarray expression data for cumulus granulosa cells from the EmbryoGENE dataset. In this dataset, the EA stage is represented by the GV0 chromatin configuration, whereas the MA stage comprises the GV1, GV2, and GV3 chromatin configurations [34, 42, 43]. The expression of ligands and receptors identified in EA and MA was evaluated by comparing normalized microarray expression intensities, reflecting relative transcript abundance, between cumulus granulosa cells from the GV0 group and those from the combined GV1–GV3 groups using a two‐sample Student's *t*‐test for independent samples.

In a subsequent analysis, ligands and receptors that were significantly differentially expressed between BCB‐positive and BCB‐negative cumulus granulosa cells were further investigated. According to a previous study, BCB‐negative oocytes are mainly associated with Class 1 COCs, characterized predominantly by the GV1 chromatin configuration, whereas BCB‐positive oocytes are generally associated with Class 2/3 COCs, mainly characterized by the GV2 configuration [34]. Based on this evidence, gene expression levels were compared between the GV1 and GV2 groups of the EmbryoGENE dataset, with GV1 considered representative of the BCB‐negative stage and GV2 of the BCB‐positive stage. Statistical differences in normalized microarray expression intensities between GV1 and GV2 cumulus granulosa cells were assessed using a two‐sample Student's *t*‐test for independent samples.

## 3 Results

### 3.1 Ligand‐Receptor Interactions in SF Identify Signaling and Metabolic Pathways

**FIGURE 2:**
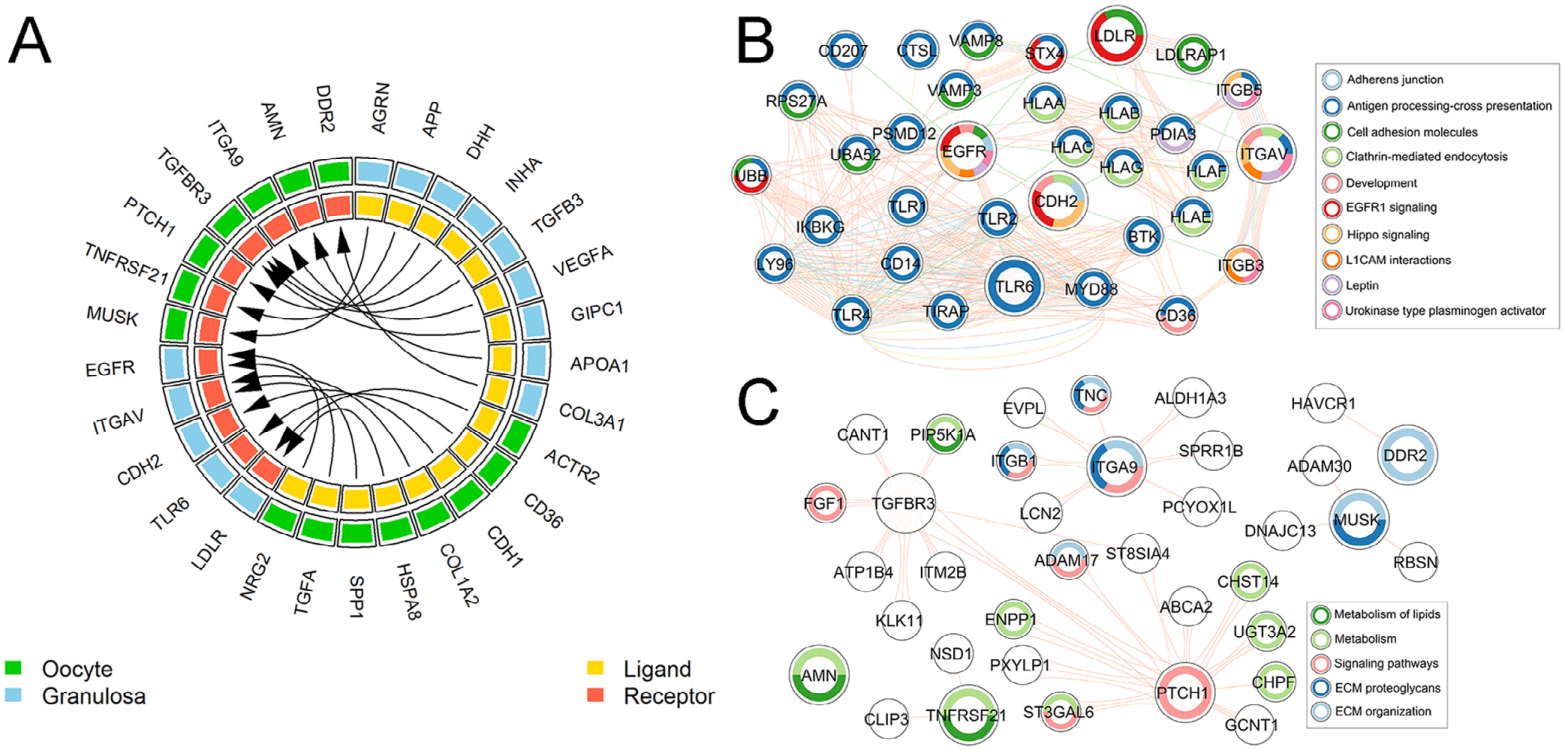
Ligand receptor interactions in SF between SFGC and SFO. (A) Chord plot illustrating interactions between ligands (yellow sectors) and receptors (orange sectors) expressed in SFGC (blue sectors) and SFO (green sectors). (B) Network of receptors expressed in SFGC connected to associated genes and downstream processes. (C) Network of receptors expressed in SFO connected to associated genes and downstream processes. In networks B and C, edges are colored according to the type of interaction: Physical interactions (orange), genetic interactions (green), pathway (blue), and predicted (yellow). The size of the nodes depends on whether the gene is a receptor (bigger circles), or a receptor‐related gene (smaller circles).

After inferring LRPs using BulkSignalR and excluding those specific to SFO‐SFO and SFGC‐SFGC communication, a total of 158 LRPs were identified. Among these, 17 LRPs showed significant differential expression between SFO and SFGC (Figure 2A).

According to BulkSignalR's correlation analysis, *INHA‐TGFBR3* showed the highest correlation (*R* = 0.86), and *AGRN‐MUSK, DHH‐PTCH1, GIPC1‐TGFBR3*, and *TGFA‐EGFR* exhibited strong correlations (*R* = 0.60–0.79). The remaining LRPs showed moderate correlations (*R* = 0.4–0.59) (File S1).

Receptors expressed in SFGC were primarily associated with antigen processing–cross presentation and cell adhesion, including *TLR6*, *ITGAV*, and *LDLR*. In addition, *ITGAV* and *LDLR*, together with *EGFR* and *CDH2*, were involved in signaling pathways related to cell proliferation and migration, with enrichment of pathways including EGFR1, Hippo, and Urokinase‐type plasminogen activator signaling. *ITGAV* and *EGFR* were also associated with leptin‐related pathways, suggesting a role for SFO‐to‐SFGC communication in metabolic regulation (Figure 2B).

The downstream pathways associated with SFO receptors encompassed several signaling and metabolic processes (Figure 2C). In particular, *PTCH1* was involved in signaling pathways, including Hedgehog signaling. Other SFO receptors, including *ITGA9*, *MUSK*, and *DDR2*, were mainly associated with ECM organization and ECM proteoglycans‐related pathways, whereas *TNFRSF21* and *AMN* were implicated in lipid metabolism. No significant functional enrichment was observed for *TGFBR3* in this analysis; however, this receptor is a well‐established component of the TGF‐beta signaling pathway, consistent with its interaction with the ligand *INHA*.

### 3.2 EAO‐EAcGC Communication Is Associated With Developmental and Metabolic Signaling

**FIGURE 3:**
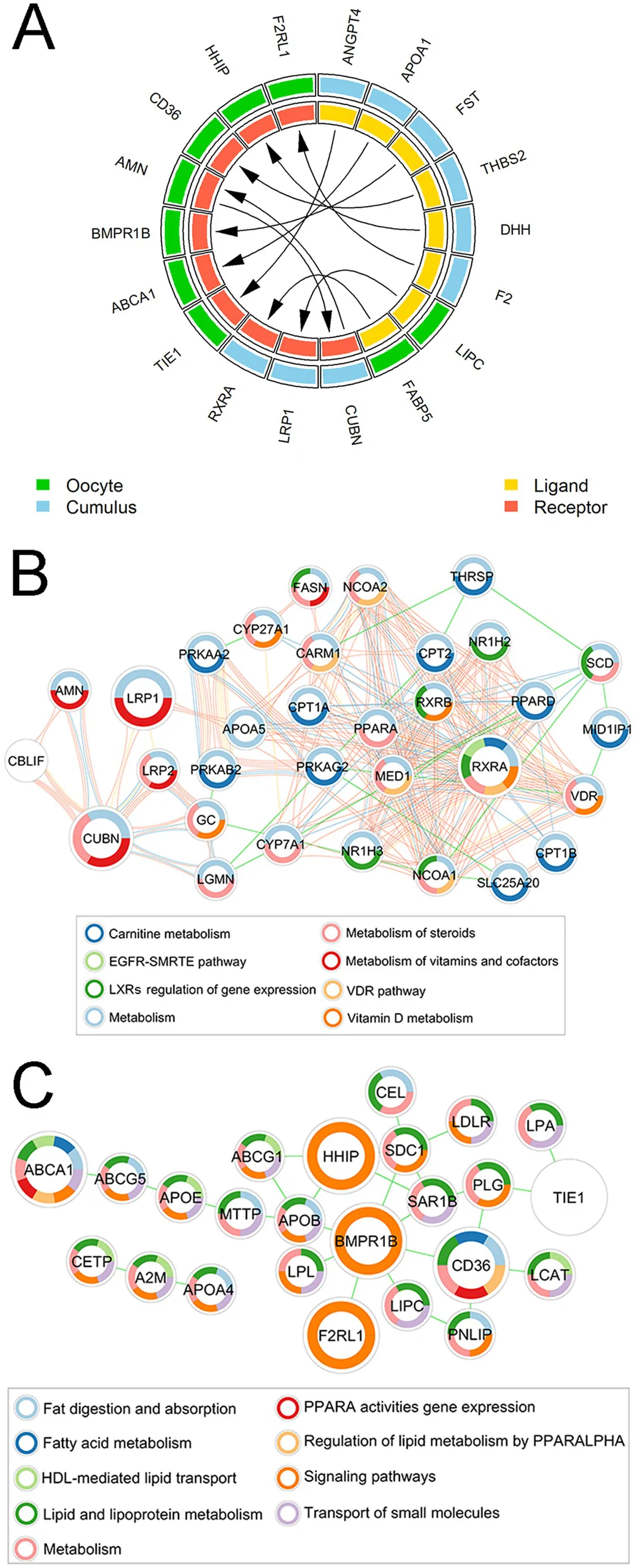
Ligand receptor interactions in EA between EAcGC and EAO. (A) Chord plot illustrating interactions between ligands (yellow sectors) and receptors (orange sectors) expressed in EAcGC (blue sectors) and EAO (green sectors). (B) Network of receptors expressed in EAcGC connected to associated genes and downstream processes. (C) Network of receptors expressed in EAO connected to associated genes and downstream processes. In networks B and C, edges are colored according to the type of interaction: Physical interactions (orange), genetic interactions (green), pathway (blue), and predicted (yellow). The size of the nodes depends on whether the gene is a receptor (bigger circles), or a receptor‐related gene (smaller circles).

After inferring LRP with BulkSignalR and excluding those specific to the EAO‐EAO and EAcGC‐EAcGC communication, a total of 122 LRPs were identified. Among these, 10 LRPs showed significant differential expression between EAO and EAcGC, including *AMN‐CUBN* interaction that works in concert for vitamin uptake [44] (Figure 3A).

BulkSignalR correlation analysis revealed that *DHH‐HHIP, FST‐BMPR1B, THBS2‐CD36, APOA1‐ABCA1, and LIPC‐LRP1* showed the strongest correlations (*R* ≥ 0.80), *FABP5‐RXRA* exhibited a very strong correlation (*R* = 0.60–0.79), whereas the remaining LRPs exhibited moderate correlations (*R* = 0.4–0.59) (File S1).

The EAcGC receptors identified among the significant LRPs, including *LRP1*, *RXRA*, and *CUBN*, were predominantly associated with vitamin metabolism, while *CUBN* and *RXRA* were also involved in steroid metabolism. Furthermore, *RXRA* was implicated in EGFR‐SMRTE signaling, which regulates cell proliferation, differentiation, and apoptosis, as well as in LXRs‐mediated gene expression regulation (Figure 3B).

The EAO receptors *HHIP, BMPR1B*, *F2RL1*, and *ABCA1* were primarily associated with signaling pathways. Consistent with their interactions with the corresponding ligands, *HHIP* was linked to Hedgehog signaling, *BMPR1B* to the TGF‐beta superfamily, *F2RL1* functioned as a G protein‐coupled receptor activated by trypsin and trypsin‐like proteases, and *ABCA1* acted as an ATP‐dependent transporter involved in lipoprotein transport. Additionally, *CD36* was mainly implicated in cell adhesion (Figure 3C).

### 3.3 Stage‐Specific Ligand‐Receptor Signaling in MA Supports Oocyte Growth and Cumulus Granulosa Cells Energy Metabolism

**FIGURE 4:**
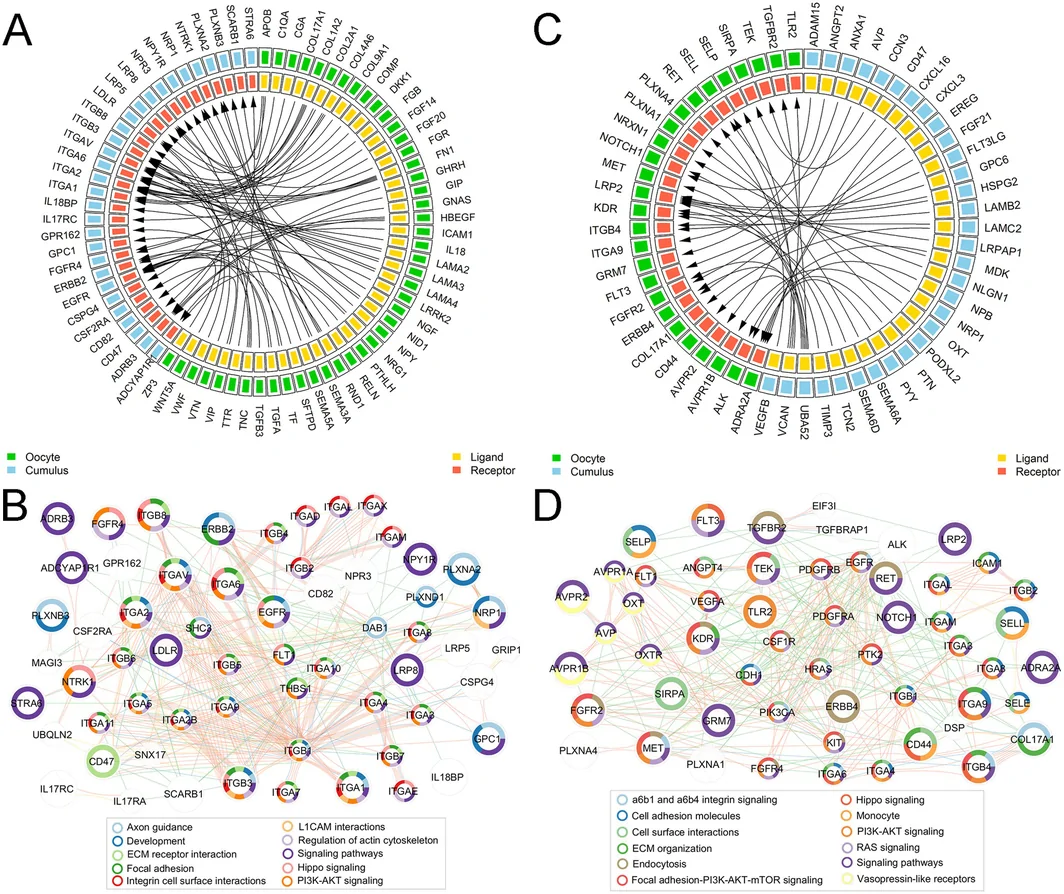
Ligand receptor interactions between MAcGC and MAO. (A) Chord plot illustrating interactions between ligands (yellow sectors) expressed in MAO (green sectors) and receptors (orange sectors) expressed in MAcGC (blue sectors). (B) Network of receptors expressed in MAcGC connected to associated genes and downstream processes. (C) Chord plot illustrating interactions between ligands (yellow sectors) expressed in MAcGC (blue sectors) and receptors (orange sectors) expressed in the MAO (green sectors). (D) Network of receptors expressed in MAcGC connected to associated genes and downstream processes. In networks B and D, edges are colored according to the type of interaction: Physical interactions (orange), genetic interactions (green), pathway (blue), and predicted (yellow). The size of the nodes depends on whether the gene is a receptor (bigger circles), or a receptor‐related gene (smaller circles).

Because no bulk RNA‐seq datasets from intact bovine middle antral follicles are currently available, ligand‐receptor interactions at this stage could not be inferred using the same BulkSignalR workflow applied to SF and EA. Instead, paired transcriptomes from MAO and their corresponding MAcGC were analyzed. The BulkSignalR ligand‐receptor database was retained as the reference set of curated ligand‐receptor pairs, whereas candidate interactions were inferred using a correlation‐based approach. A correlation model was employed, and 320 LRPs between the MAcGC ligand and the MAO receptor and 341 LRPs between the MAcGC receptor and the MAO ligand were identified. Among these LRPs, 42 and 72, respectively, involved ligands and/or receptors that were significantly differentially expressed between MAcGC and MAO (Figure 4A,C). Among the inferred interactions, several well‐characterized LRPs were identified, including GDF9‐BMPR2, BMP15‐BMPR2, INHA‐ACVR2A, INHA‐ACVR1, INHBB‐ACVR2B, and INHBB‐ACVR2A, supporting the biological validity of the approach [24, 45, 46, 47]. Moreover, similar LRPs were found to those in a previous study [24], such as *TEK‐ANGPT4* and members of the Plexin family with *SEMA* and *RND1* ligands.

Functional enrichment analysis of genes associated with MAcGC receptors revealed involvement in several biological processes, including axon guidance and major signaling pathways, such as PI3K‐AKT. Notably, enrichment of mechanisms related to other signaling pathways, such as endocytosis (*LDLR, LRP8*), G protein‐coupled receptor activity (*NPY1R, ADRB3, ADCYAP1R1*), and lipid and vitamin metabolism (*FGFR4, STRA6*), was observed. In addition, multiple interactions involving focal adhesion and integrins emerged as a prominent feature of the network, as evidenced by the high representation of integrin genes (Figure 4B).

The downstream network and pathway related to the MAO receptors highlighted enrichment of the well‐established PI3K‐AKT, mTOR, and RAS signaling pathways. Moreover, cell adhesion molecules and ECM organization were present. Also, other signaling pathways, such as Hippo (*FGFR2, FLT3, TEK, ITGB4, KDR, ITGA9, MET, CD44*), NOTCH (*NOTCH1*), TGF‐beta (*TGFBR2*), and receptors part of the G protein‐coupled receptors family (*AVPR1B, AVPR2, GRM7, ADRA2A*), were involved in MAO receptor downstream signaling (Figure 4D).

Table 2 list the LRPs with the strongest correlation (*R* > 0.6) between (A) MAcGC receptors and MAO ligands, and (B) MAcGC ligands and MAO receptors. All LRPs with significant positive correlations are reported in File S2.

**TABLE 2:** Inferred ligand‐receptor pairs in the MA that show a significant positive Pearson correlation between ligands and receptors.

Ligand in MAO	Receptor in MAcGC	Differential expression between MAcGC‐pos vs MAcGC‐neg (log2FC; *p*‐value)	Correlation between ligand and receptor (*R*)
A			
*RND1* (Rho Family GTPase 1)	*PLXNA2* (Plexin A2)	1.7261; **	0.795
*COL2A1* (Collagen Type II Alpha 1 Chain)	*ITGA2* (Integrin Subunit Alpha 2)	1.5706; **	0.791
*LAMA4* (Laminin Subunit Alpha 4)	*ITGA6* (Integrin Subunit Alpha 6)	1.599; **	0.785
*COL1A2* (Collagen Type I Alpha 2 Chain)	*ITGB3* (Integrin Subunit Beta 3)	1.4356; **	0.764
*COL4A6* (Collagen Type IV Alpha 6 Chain)	*CD47* (CD47 Molecule)	1.5134; **	0.763
*FN1* (Fibronectin 1)	*ITGB3* (Integrin Subunit Beta 3)	1.4356; **	0.751
*VIP* (Vasoactive Intestinal Peptide)	*ADCYAP1R1* (ADCYAP Receptor Type I)	0.3085; ns	0.736
*LAMA4* (Laminin Subunit Alpha 4)	*ITGAV* (Integrin Subunit Alpha V)	1.2262; *	0.719
*FN1* (Fibronectin 1)	*ITGA6* (Integrin Subunit Alpha 6)	1.599; **	0.713
*FN1* (Fibronectin 1)	*ITGA2* (Integrin Subunit Alpha 2)	1.5706; **	0.704
*FN1* (Fibronectin 1)	*IL17RC* (Interleukin 17 Receptor C)	1.0402; *	0.701
*COL1A2* (Collagen Type I Alpha 2 Chain)	*ITGA2* (Integrin Subunit Alpha 2)	1.5706; **	0.694
*ZP3* (Zona Pellucida Glycoprotein 3)	*EGFR* (Epidermal Growth Factor Receptor)	1.7542; ***	0.688
*LAMA3* (Laminin Subunit Alpha 3)	*ITGA2* (Integrin Subunit Alpha 2)	1.5706; **	0.686
*CGA* (Glycoprotein Hormones, Alpha Polypeptide)	*ADRB3* (Adrenoceptor Beta 3)	0.302; ns	0.679
*HBEGF* (Heparin Binding EGF Like Growth Factor)	*CD82* (CD82 Molecule)	1.4392; **	0.675
*LAMA3* (Laminin Subunit Alpha 3)	*ITGA6* (Integrin Subunit Alpha 6)	1.599; **	0.662
*SFTPD* (Surfactant Protein D)	*CSF2RA* (Colony Stimulating Factor 2 Receptor Subunit Alpha)	1.1345; *	0.661
*NGF* (Nerve Growth Factor)	*NTRK1* (Neurotrophic Receptor Tyrosine Kinase 1)	1.9548; **	0.658
*COL4A6* (Collagen Type IV Alpha 6 Chain)	*ITGA2* (Integrin Subunit Alpha 2)	1.5706; **	0.656
*APOB* (Apolipoprotein B)	*LDLR* (Low Density Lipoprotein Receptor)	1.6751; **	0.652
*HBEGF* (Heparin Binding EGF Like Growth Factor)	*EGFR* (Epidermal Growth Factor Receptor)	1.7542; ***	0.650
*SEMA3A* (Semaphorin 3A)	*PLXNA2* (Plexin A2)	1.7261; **	0.642
*ICAM1* (Intercellular Adhesion Molecule 1)	*EGFR* (Epidermal Growth Factor Receptor)	1.7542; ***	0.633
*LAMA2* (Laminin Subunit Alpha 2)	*ITGA6* (Integrin Subunit Alpha 6)	1.599; **	0.631
*COL1A2* (Collagen Type I Alpha 2 Chain)	*ITGA1* (Integrin Subunit Alpha 1)	1.6789; **	0.628
*GIP* (Gastric Inhibitory Polypeptide)	*ADRB3* (Adrenoceptor Beta 3)	0.3020; ns	0.625
*COL2A1* (Collagen Type II Alpha 1 Chain)	*ITGA1* (Integrin Subunit Alpha 1)	1.6789; **	0.606
*DKK1* (Dickkopf Wnt Signaling Pathway Inhibitor 1)	*LRP5* (LDL Receptor Related Protein 5)	1.354; **	0.601

Ligand in MAcGC	Receptor in MAO	Differential expression between MAcGC‐pos vs MAcGC‐neg	Correlation between ligand and receptor (*R*)
B			
*UBA52* (Ubiquitin A‐52 Residue Ribosomal Protein Fusion Product 1)	*FGFR2* (Fibroblast Growth Factor Receptor 2)	1.4382; **	0.689
*TIMP3* (TIMP Metallopeptidase Inhibitor 3)	*KDR* (Kinase Insert Domain Receptor)	1.283; *	0.678
*UBA52* (Ubiquitin A‐52 Residue Ribosomal Protein Fusion Product 1)	*LRP2* (LDL Receptor Related Protein 2)	1.4382; **	0.671
*UBA52* (Ubiquitin A‐52 Residue Ribosomal Protein Fusion Product 1)	*MET* (MET Proto‐Oncogene, Receptor Tyrosine Kinase)	1.4382; **	0.668
*GPC6* (Glypican‐6)	*LRP2* (LDL Receptor Related Protein 2)	1.4874; **	0.655
*VCAN* (Versican)	*SELP* (Selectin P)	1.2683; **	0.630
*MDK* (Midkine)	*LRP2* (LDL Receptor Related Protein 2)	1.3564; **	0.625
*CD47* (CD47 Molecule)	*SIRPA* (Signal Regulatory Protein Alpha)	1.5134; **	0.625
*HSPG2* (Heparan Sulfate Proteoglycan 2)	*KDR* (Kinase Insert Domain Receptor)	1.2961; **	0.621
*LRPAP1* (LDL Receptor Related Protein Associated Protein 1)	*LRP2* (LDL Receptor Related Protein 2)	1.9081; **	0.615
*LAMC2* (Laminin Subunit Gamma 2)	*COL17A1* (Collagen Type XVII Alpha 1 Chain)	1.6227; **	0.614
*EREG* (ETS Transcription Factor ERG)	*ERBB4* (Erb‐B2 Receptor Tyrosine Kinase 4)	0.6683; ns	0.611

*Note:* (A) Ligands in MAO and corresponding MAcGC receptors. (B) Ligands in MAcGC and corresponding MAO receptors. *R* indicates the strength and direction of the linear relationship between ligand and receptor expression. Differential expression of ligands or receptors in cumulus granulosa cells according to BCB staining status was estimated as the log2 fold change (log2FC), calculated as the difference between the mean expression levels of MAcGC‐pos and MAcGC‐neg samples. Statistical significance was assessed using one‐way ANOVA. log2FC: log2 fold change; *p*‐values: *p < 0.05, **p < 0.01, ***p < 0.001; *R*: Pearson correlation coefficient.

During the MA stage, distinct populations of COCs can be identified [42, 48, 49]. One approach to account for this heterogeneity is the use of BCB dye. Focusing exclusively on the LRPs identified above, we found that 47 MAcGC ligand and receptor genes were differentially expressed between BCB‐positive and BCB‐negative samples (File S3). Among these, 12 receptors and 9 ligands (Table 2) also displayed strong correlation with the corresponding oocyte ligand or receptor. In contrast, *NGF* was the only gene identified within the LRPs that was differentially expressed between MAO‐pos and MAO‐neg samples (File S3).

### 3.4 cGC Ligands and Receptors Show Stage‐Specific Expression Patterns According to Oocyte Chromatin Configuration

**FIGURE 5:**
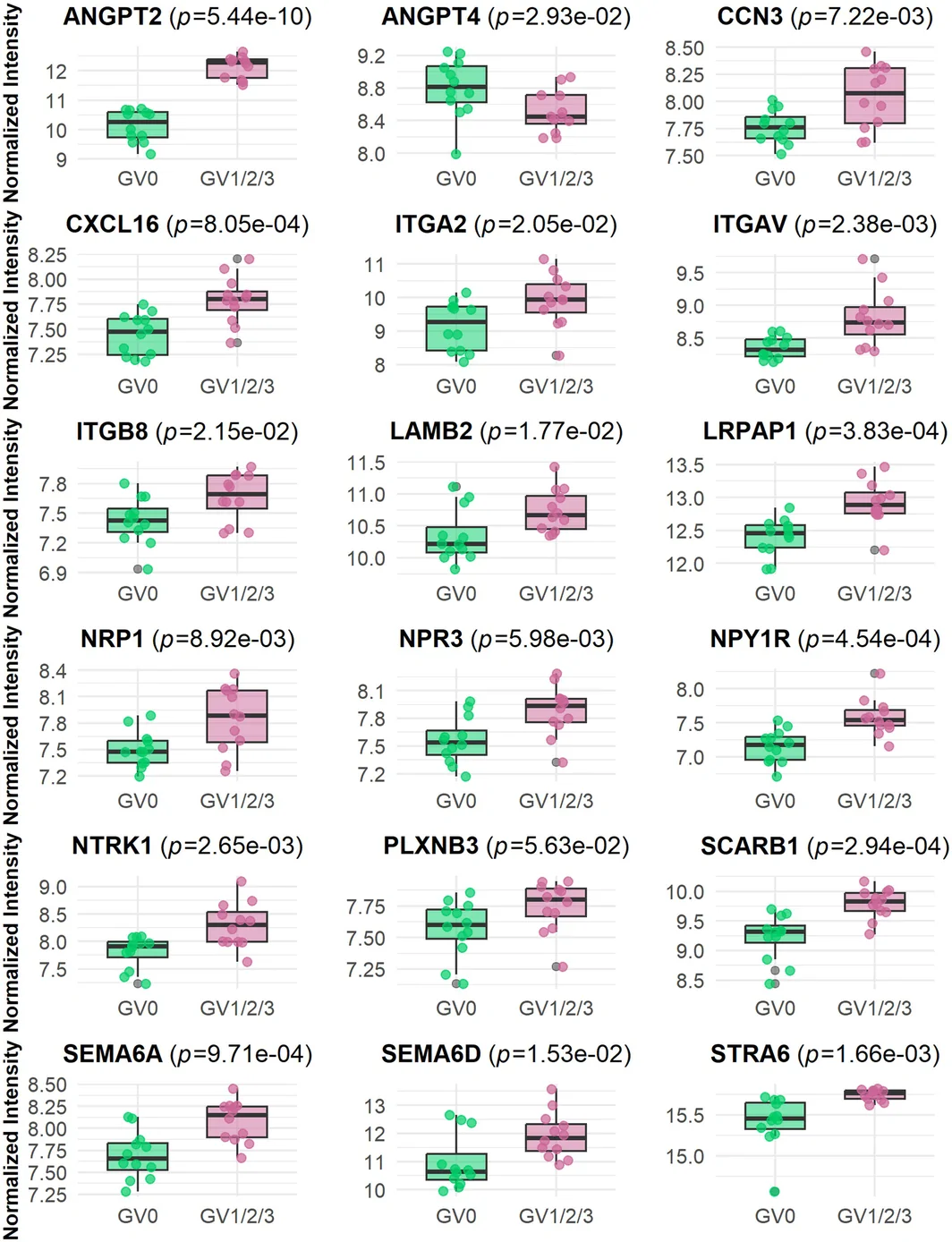
Validation of EAcGC‐ and MAcGC‐associated ligand and receptor expression. Ligands and receptors identified for the EAcGC and MAcGC were further validated for differential expression using the EmbryoGENE dataset, in which follicular stages are also classified based on chromatin conformation. In this analysis, the EA stage was defined as GV0, whereas the MA stage comprised GV1, GV2, and GV3. Box plots represent the normalized gene expression intensity measured by microarray. The *p*‐value for the comparison between the EA and MA stages is reported above each plot. The figure shows genes that are either significantly differentially expressed (*p* < 0.05) or display a trend toward significance (*p* = 0.05–0.1).

To validate the stage specificity of ligand and receptor expression in EAcGC and MAcGC, we used the EmbryoGENE dataset. Among the genes identified in our analysis, the EAcGC‐expressed ligand *ANGPT4* was significantly differentially expressed between the cumulus cells of the EA and MA groups. Furthermore, 16 ligands and receptors identified in MAcGC were significantly differentially expressed between the EA and MA groups (*p* < 0.05), while *PLXNB3* showed a trend toward significance (*p* = 0.05–0.1) (Figure 5).

**FIGURE 6:**
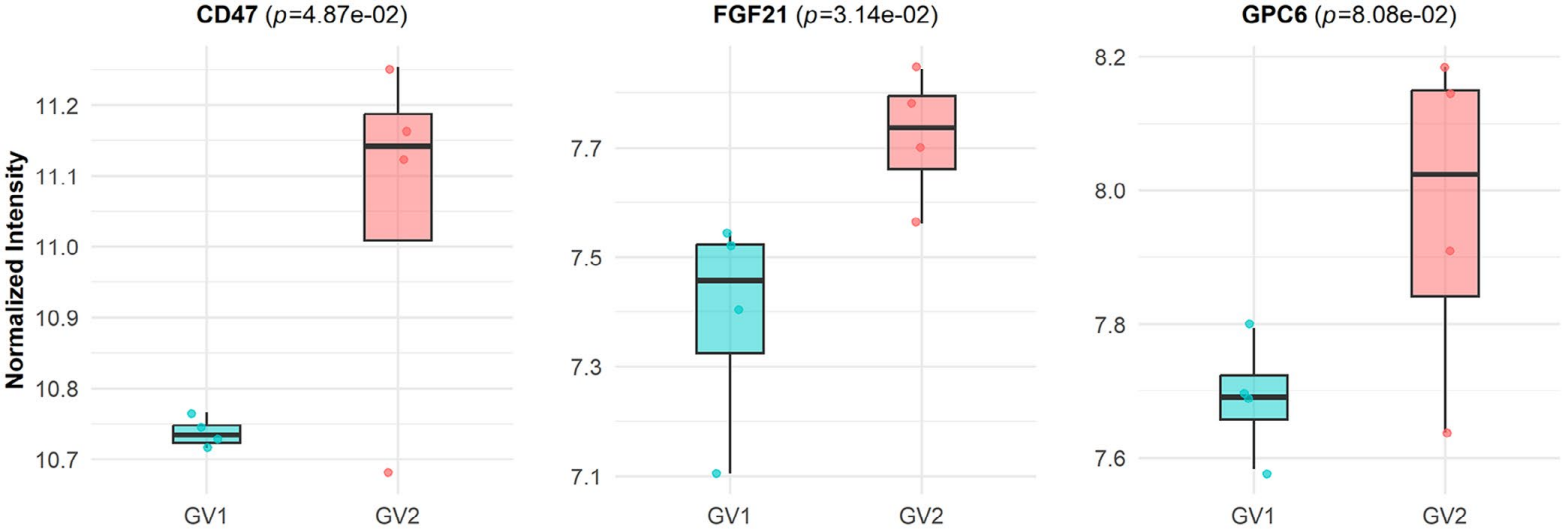
Validation of BCB‐associated ligand and receptor expression within the MA stage. Within the MA stage, ligand and receptor genes previously identified as significantly differentially expressed by BCB staining were further analyzed using the EmbryoGENE dataset by comparing GV1 versus GV2 stages. The figure shows genes that are either significantly differentially expressed (*p* < 0.05) or display a trend toward significance (*p* = 0.05–0.1). Box plots represent normalized microarray expression intensity, and the *p* for each comparison is reported above the corresponding plot.

To determine whether the BCB‐dependent differences observed in MAcGC were associated with oocyte chromatin configuration, ligands and receptors that were differentially expressed between MAcGC‐pos and MAcGC‐neg samples were further examined in the EmbryoGENE dataset by comparing the expression profile of cumulus cells derived from GV1 and GV2 stages. *CD47* and *FGF21* were significantly differentially expressed across chromatin configuration stages (*p* < 0.05), consistent with the pattern observed following BCB classification. *GPC6* showed a similar trend, although it did not reach statistical significance (*p* = 0.05–0.1) (Figure 6).

## 4 Discussion

**FIGURE 7:**
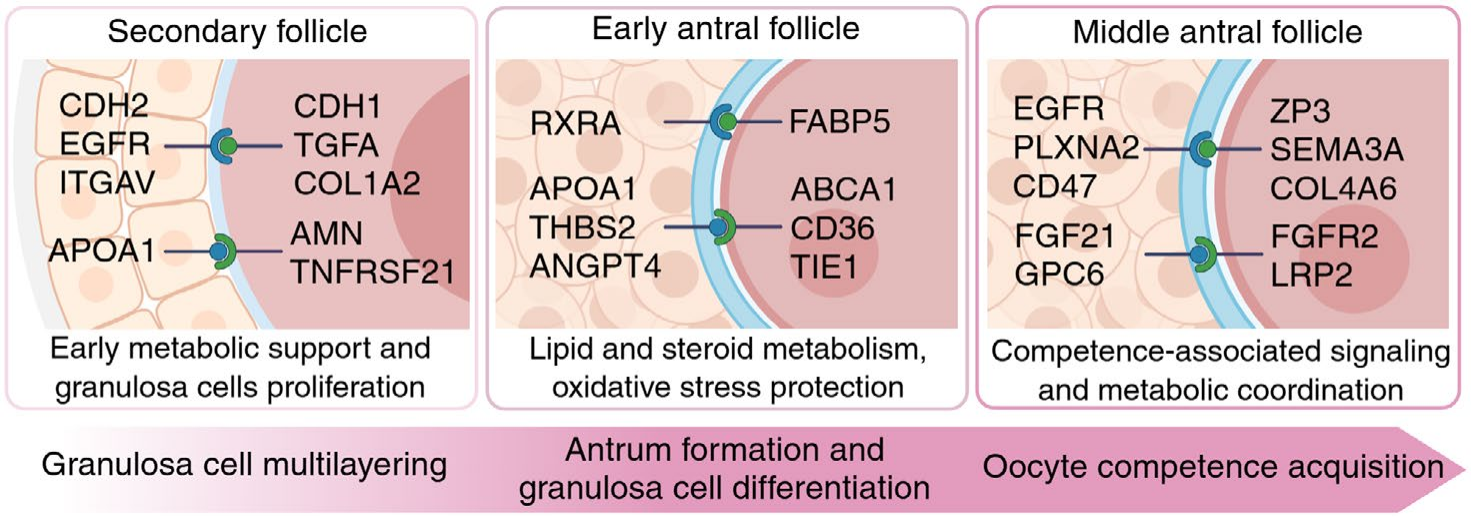
Schematic representation of the ligand‐receptor interactions identified across the secondary, early antral, and middle antral follicle stages, showing regulatory processes associated with oocyte development. Ligands expressed by granulosa cells are depicted as blue circles, whereas ligands expressed by the oocyte are shown as green circles. Receptors are represented by blue and green semicircles, corresponding to granulosa cells and the oocyte, respectively. The main signaling pathways involved at each developmental stage are indicated below the scheme. The arrow with an increasing color gradient illustrates the progressive molecular and cellular changes occurring during follicular growth within each follicular stage. Created in BioRender. Monferini, N. (2026) https://BioRender.com/8vyaj8y.

Bidirectional communication between the oocyte and surrounding granulosa cells is now recognized as the fundamental mechanism coordinating folliculogenesis, integrating the developmental programs of both the germinal and somatic compartments. Rather than functioning as a unidirectional support system, follicle development relies on continuous reciprocal signaling, with granulosa cells providing metabolic and structural support to the oocyte while the oocyte actively regulates granulosa cell proliferation, differentiation, and function [50, 51]. Within this conceptual framework, the seminal studies of Eppig and colleagues [52] established that the oocyte is an active regulator of granulosa cell behavior, fundamentally reshaping our understanding of follicular biology. Consistent with this model of reciprocal communication, our findings indicate that germinal–somatic signaling is progressively remodeled during folliculogenesis (Figure 7), evolving from pathways associated with structural organization and metabolic support in secondary follicles toward increasingly complex signaling networks accompanying the acquisition of developmental competence.

One of the principal findings emerging from this study is the progressive metabolic specialization of oocyte–granulosa cell communication throughout folliculogenesis. Although metabolic cooperation was evident at every developmental stage, the nature of the associated signaling pathways evolved considerably as follicles matured. Receptors associated with lipid metabolism were identified throughout folliculogenesis, highlighting the continuous metabolic interdependence between the germinal and somatic compartments. In secondary follicle oocytes, *AMN* and *TNFRSF21*, and in early antral follicle oocytes, *ABCA1* and *CD36*, were associated with lipid‐related pathways. *AMN* and *ABCA1* interact with *APOA1*, which is highly expressed in granulosa cells and contributes to lipid transport and substrate availability for energy production and steroidogenesis [53, 54, 55, 56, 57, 58]. Together, these observations indicate that metabolic cooperation is established early during folliculogenesis and progressively adapts to the increasing energetic and biosynthetic demands of the growing follicle [59].

In secondary follicles, the identified ligand‐receptor interactions were mainly associated with cell adhesion, extracellular matrix organization, and early metabolic regulation. Specifically, *CDH2* emerged as a central component of germinal‐somatic compartments communication in secondary follicles. Cadherins are known to maintain physical interactions between the oocyte and granulosa cells, preserve granulosa cell architecture, and support the formation of multiple granulosa cell layers, all of which are fundamental for proper follicle organization [60, 61, 62]. In addition, receptors associated with EGFR and Hippo signaling pathways suggest mechanisms underlying granulosa cell proliferation, polarity, and tissue remodeling [63, 64, 65]. Metabolic signaling was also evident at this stage, including pathways associated with leptin signaling. Previous studies have demonstrated beneficial effects of leptin on preantral follicle development by regulating energy homeostasis, mitochondrial function, and oxidative stress [55, 56, 57, 58], supporting a potential role for this pathway in early folliculogenesis.

The early antral stage represents a major transition in follicular development, characterized by antrum formation, granulosa cell differentiation, and increasing responsiveness to endocrine signals [2]. Consistent with these biological changes, our analyses revealed substantial enrichment of pathways related to lipid, steroid, and vitamin metabolism. Tight regulation of lipid metabolism is particularly important during this stage because excessive accumulation of fatty acids can compromise oocyte quality and induce lipotoxicity within the cumulus‐oocyte complex [66, 67, 68]. Among the identified receptors, *RXRA* emerged as a potentially relevant component of oocyte‐granulosa cell communication in early antral follicles. *RXRA* participates in vitamin D signaling and steroid‐related pathways, both of which have been implicated in follicular development, steroidogenesis, and the regulation of oxidative stress [69, 70]. Together with pathways involving PPAR signaling and carnitine metabolism [71, 72, 73, 74, 75, 76], these findings suggest that the EA stage represents a metabolic transition phase in which multiple regulatory systems converge to support continued follicular growth while maintaining metabolic homeostasis.

The marked expansion of ligand‐receptor interactions observed in middle antral follicles suggests that acquisition of developmental competence is accompanied by extensive remodeling of intercellular signaling networks. This observation is consistent with the extensive coordination required to synchronize nuclear, cytoplasmic, and metabolic maturation before ovulation. Functional enrichment analyses revealed that the inferred ligand‐receptor network converged on signaling pathways regulating cell survival, proliferation, metabolism, extracellular matrix remodeling, and cell–cell communication, including the PI3K‐AKT, mTOR, RAS, Hippo, and TGF‐beta pathways [77, 78, 79, 80, 81, 82, 83, 84, 85, 86, 87]. Together, these findings support the concept that bidirectional communication between the germinal and somatic compartments becomes progressively more integrated as folliculogenesis advances.

In addition to these canonical signaling pathways, receptors expressed in cumulus granulosa cells of middle antral follicles were enriched for functions related to axon guidance, integrin signaling, and G protein‐coupled receptor activity. Although axon guidance mechanisms are classically associated with neuronal development [88, 89], similar molecular mechanisms have been proposed to regulate transzonal projection formation between cumulus cells and the oocyte [24]. These projections are essential for the transfer of metabolites and signaling molecules that maintain oocyte meiotic arrest and coordinate maturation [19, 42, 90]. Likewise, the enrichment of integrin‐mediated interactions, particularly the subunits α1, α2, α6, αV, β3, and β8, further highlights the importance of extracellular matrix remodeling and cell adhesion during the acquisition of oocyte competence [91, 92, 93, 94].

Finally, the enrichment of receptors involved in lipid transport, nutrient utilization, and energy metabolism further emphasizes the strong metabolic interdependence between the oocyte and cumulus granulosa cells [16, 95, 96, 97, 98, 99, 100]. Because many of the corresponding ligands originated from the oocyte, these findings are consistent with previous evidence indicating that the oocyte actively regulates the metabolic activity of the surrounding cumulus cells, thereby contributing to the establishment of a microenvironment that supports developmental competence.

To further explore whether ligand‐receptor expression patterns were associated with oocyte developmental status, we validated cumulus‐cell‐derived ligands and receptors using the independent EmbryoGENE dataset. Among the identified candidates, *ANGPT4* emerged as the only ligand specifically associated with the early antral stage. *ANGPT4* belongs to the angiopoietin family and has been implicated in vascular remodeling, angiogenesis, and follicular function [101, 102, 103, 104]. Although its role in bovine folliculogenesis remains poorly characterized, its specific expression pattern suggests a potential contribution to antral follicle development and the establishment of the follicular microenvironment [105, 106].

Additional analyses focusing on developmental competence identified several ligand and receptor genes that were more highly expressed in BCB‐positive cumulus granulosa cells of middle antral follicles. Many of these molecules were linked to pathways associated with PI3K‐AKT, mTOR, RAS, cell adhesion, and signal transduction, suggesting that developmentally competent cumulus‐oocyte complexes may exhibit enhanced communication capacity between the somatic and germinal compartments.

Among the competence‐associated candidates, *CD47*, *FGF21*, and *GPC6* were consistently supported by both the BCB‐based and chromatin‐configuration‐based analyses. These genes encode molecules directly involved in oocyte–cumulus–granulosa cell communication in middle antral follicles, with *FGF21* and *GPC6* acting as ligands and *CD47* functioning as a receptor. In particular, upregulated *CD47* is a marker of ovarian cancer [107]. However, at normal levels, as a surface receptor, it can influence downstream pathways such as PI3K/AKT and MAPK/ERK, modulating cellular functions, including metabolism, stress responses, cell death, and cell proliferation [108]. Although the present study does not establish causal relationships, their expression patterns suggest potential involvement in the acquisition of developmental competence. *FGF21* may support FGFR2‐mediated signaling in the oocyte, potentially influencing pathways such as Ras–MAPK and PI3K–AKT that are known regulators of oocyte competence [109, 110]. Similarly, *GPC6*, a member of the glypican family, a group of molecules expressed in cancers and embryonic tissue during development [111], was predicted to interact with *LRP2*, a receptor implicated in lipid uptake and metabolic regulation [112, 113]. Given the importance of lipid metabolism during oocyte growth and early embryogenesis [114, 115], the GPC6–LRP2 interaction is a promising candidate pathway for future functional investigation. Finally, these genes showed higher expression in cumulus granulosa cells of BCB‐positive oocytes isolated from middle antral follicles and in cumulus cells associated with GV2 oocytes, suggesting that they may represent molecular markers of a more advanced, developmentally competent middle antral COC subpopulation.

An important strength of this study lies in the use of multiple independent transcriptomic datasets encompassing distinct stages and cellular compartments of bovine folliculogenesis. Rather than relying on a single experimental model, the analytical framework combines datasets generated by different laboratories and further evaluates stage‐ and competence‐associated expression patterns using the independent EmbryoGENE resource. Confidence in the inferred communication networks is additionally supported by the recovery of several canonical ligand–receptor interactions previously demonstrated experimentally, including GDF9–BMPR2, BMP15–BMPR2, and members of the inhibin/activin signaling pathways. Together, these complementary levels of evidence support the biological plausibility of the identified networks and provide a robust basis for prioritizing novel candidate interactions (Figure 7).

Several limitations should nevertheless be acknowledged. First, the absence of suitable transcriptomic datasets from isolated bovine oocytes and granulosa cells at the primordial and primary follicle stages prevented extension of the analysis to the earliest phases of folliculogenesis. However, the focus on secondary and antral follicles enabled a detailed investigation of the developmental interval during which follicular organization, metabolic specialization, and oocyte competence progressively emerge. Second, transcript abundance alone cannot establish ligand secretion, receptor activation, or downstream functional activity. This limitation is particularly relevant in the oocyte, where transcription and translation may be temporally uncoupled for a subset of maternal transcripts [51]. Accordingly, the inferred ligand–receptor pairs should be regarded as biologically supported candidate interactions requiring future validation at the protein and functional levels. Finally, although the integration of independent datasets increases robustness and reduces study‐specific bias, differences in experimental design, sequencing platforms, and sample composition may limit the detection of interactions that are not consistently represented across datasets.

Within these boundaries, the present study provides a systematic transcriptomic framework describing how oocyte–granulosa cell communication progresses from structural organization and early metabolic cooperation in secondary follicles toward increasingly complex signaling networks associated with developmental competence in middle antral follicles.

## 5 Conclusions

This study provides a transcriptomic framework describing the dynamic remodeling of candidate ligand–receptor‐mediated communication between oocytes and granulosa cells during bovine folliculogenesis. The analyses reveal a developmental progression from interactions supporting structural organization and early metabolic cooperation in secondary follicles to increasingly complex signaling networks accompanying metabolic specialization and the acquisition of developmental competence in antral follicles. In addition to recovering established communication pathways, the study prioritizes candidate regulators, including *CD47*, *FGF21*, and *GPC6*, for future functional investigation. Collectively, these findings provide a biologically grounded resource for advancing the study of follicular development and informing the rational improvement of in vitro follicle and cumulus‐oocyte complex culture systems.

## Author Contributions

N.M. conceptualization, data curation, formal analysis, writing – original draft preparation; L.D. data curation, writing – review and editing; P.D. data curation, writing – review and editing; F.F. writing – review and editing; V.L. writing – review and editing; M.B.R. conceptualization, data curation, supervision, writing – original draft preparation; A.M.L. conceptualization, supervision, writing – original draft preparation, funding acquisition, project administration.

## Funding

This work was supported by Regione Lombardia FEASR—Programma di Sviluppo Rurale 2014‐2020, Sottomisura 10.2—“Sostegno per la conservazione, l'uso e lo sviluppo sostenibili delle risorse genetiche in agricoltura”—Operazione 10.2.01—“Conservazione della biodiversità animale e vegetale” No. 202102146691 (R‐INNOVA), by the Italian Ministry of University and Research (MUR), PRIN2020, No. 20209L8BN4 (InfinitEGG), by European Union's Horizon 2020 Marie Skłodowska‐Curie Innovative Training Network, grant agreement No. 860960 (“EUROVA”), and from the European Union Next‐GenerationEU PIANO NAZIONALE DI RIPRESA E RESILIENZA (PNRR) Missione 4—Componente 2, Italian Ministry of University and Research MUR, PRIN2022, No. 20227EB74M (CO‐MATRIX). The PD fellowship is supported by funding from the European Union's Horizon 2020 Marie Skłodowska‐Curie Innovative Training Network, grant agreement No. 860960 (“EUROVA”); the NM fellowship is funded by the MUR Programma Operativo Nazionale (PON) “Ricerca e Innovazione” 2014–2020—REACT EU—DM1061/2021. All the above research grants were awarded to AML. NM was awarded the “Ermenegildo Zegna Founder's Scholarship 2025,” which supported a research internship that contributed to this study.

## Ethics Statement

The authors have nothing to report.

## Consent

The authors have nothing to report.

## Conflicts of Interest

The authors declare no conflicts of interest.

## Supporting information


**File S1:** SF and EA ligand‐receptor interactions between the somatic and germinal compartments. **ST1A** BulkSignalR‐identified LRPs in SF, including differentially expressed genes in SFGC and SFO, with adjusted *p*‐values and log2 fold changes (log2FC). Correlation values are reported for ligand expression in SFGC with receptor expression in SFO, and for ligand expression in SFO with receptor expression in SFGC, as computed using the BulkSignalR package. **ST1B** BulkSignalR‐identified LRPs in EA, including differentially expressed genes in EAcGC and EAO, with adjusted *p*‐values and log2 fold changes (log2FC). Correlation values are reported for ligand expression in EAcGC with receptor expression in EAO, and for ligand expression in EAO with receptor expression in EAcGC, as computed using the BulkSignalR package.
**File S2:** Ligand receptor positive correlations. **Figure S1A** MAcGC receptors positively correlated with MAO ligands. **Figure S1B** MAcGC ligands positively correlated with MAO receptors. The correlation graphs display all positive Pearson correlations; the correlation coefficient (*R*) and corresponding *p* are indicated at the top of each graph.
**File S3:** Differential gene expression of ligand and receptors based on BCB staining. Box plots show the differential expression of LRPs according to BCB staining. Expression differences are reported for A MAcGC ligands and B MAO receptors, C MAcGC receptors and D MAO ligands. Turquoise indicates expression levels in MAO‐neg and MAcGC‐neg, while purple indicates those derived from MAO‐pos and MAcGC‐pos. Statistical significance was assessed using ANOVA. Asterisks denote levels of statistical significance (*p < 0.05; **p < 0.01; ***p < 0.001).

## Data Availability

The datasets analyzed in the current study comprise publicly available datasets, as detailed and cited in Table 1, and in‐house‐generated datasets associated with independent manuscripts. All in‐house datasets are publicly available, with the exception of GSE309598, which has been deposited in GEO and will be made publicly available upon publication of the associated manuscript. The scripts used for data processing and analysis are publicly available on Zenodo (https://doi.org/10.5281/zenodo.22099204) and GitHub (https://github.com/nnoemim/LRFollicle).
