# Supplementary material for: Dynamic Remodeling of Oocyte‐Granulosa Cell Communication During Bovine Folliculogenesis Revealed by Transcriptomic Meta‐Analyses
Source: FASEB J. 2026 Sep 11;40(18):e72289. doi: 10.1096/fj.202603316RR (PMC13564240; doi:10.1096/fj.202603316RR)
Supplement: Supplementary file 1 [file FSB2-40-e72289-s001.zip › 202603316RR-sup-0004-SI_Table-S02.pdf]

Additional File 1. SF and EA ligand-receptor interactions between the somatic and germinal compartments. **ST1A** BulkSignalR-identified LRPs in SF, including differentially expressed genes in SFGC and SFO, with adjusted *p*-values and log2 fold changes (log2FC). Correlation values are reported for ligand expression in SFGC with receptor expression in SFO, and for ligand expression in SFO with receptor expression in SFGC, as computed using the BulkSignalR package. **ST1B** BulkSignalR-identified LRPs in EA, including differentially expressed genes in EAcGC and EAO, with adjusted *p*-values and log2 fold changes (log2FC). Correlation values are reported for ligand expression in EAcGC with receptor expression in EAO, and for ligand expression in EAO with receptor expression in EAcGC, as computed using the BulkSignalR package.

ST1A

BulkSignalR LRPs

Ligand	Receptor
ACTR2	LDLR
ADAM10	AXL
ADAM15	ITGB1
ADAM9	ITGA3
AGRN	ITGB1
AGRN	LRP1
AGRN	MUSK
APOE	LRP5
APOE	LRP8
APOE	SCARB1
APP	TNFRSF21
ARF1	CHRM3
ARF1	INSR
CALR	ITGAV
CD36	TLR6
CDH1	CDH2
CDH1	IGF1R
COL16A1	ITGB1
COL18A1	GPC1
COL18A1	ITGA3
COL18A1	ITGB1
COL1A1	ITGB1
COL1A2	ITGAV
COL1A2	ITGB1
COL3A1	ITGB1
COL4A1	ITGB1
COL4A3	ITGA3
COL4A3	ITGB1
COL4A4	ITGB1
COL4A6	ITGAV
COL5A1	ITGB1
COL6A2	ITGB1
DHH	CDON
DHH	PTCH1
EFNA1	EPHA5
EFNA1	EPHA6
EFNA1	EPHA7
EFNB2	EPHA6
FBN1	ITGAV
FGF2	SDC3
GDF1	ACVR2B
GNAI2	ADCY9
GNAI2	F2R
GNAS	ADCY9
GPC6	LRP1
HBEGF	ERBB4
HSPA8	LDLR
HSPG2	ITGB1
HSPG2	LRP1
IGFBP4	LRP6
IL1RN	IL1R1
INHA	ACVR2A
INHA	TGFBR3
INHBA	BAMBI
IRAK4	TLR6
ITGB3BP	ITGB3
ITGB3BP	ITGB5
JAG2	NOTCH4
LAMA1	GPC1
LAMA1	ITGA3
LAMA1	ITGB1
LAMA1	SDC2
LAMA2	ITGB4
LAMB1	ITGB1
LAMB2	ITGB1
LAMC1	ITGA3
LAMC3	ITGA2
LAMC3	ITGA6
LAMC3	ITGB1
LTBP3	ITGB5
LTB	LTBR
LTB	TNFRSF1A
LTF	TFRC
MDK	ITGA6
MDK	ITGB1
MFGE8	ITGAV
NRG4	ERBB4
PDGFC	PDGFRA
POMC	RAMP2
PROS1	AXL

Differentially expressed ligand and receptors in SFGC

gene	adj_p_value	log2FC
ITGAM	4.91009940025938e-29	-9.05885954024699
SHBG	1.01712476095014e-25	-9.4613426672667
VCAM1	4.19850079496705e-24	-8.92329195313769
INHA	1.25941289637304e-15	-14.8165947928129
COL18A1	3.13467426677506e-14	-12.2814724152782
APOA1	6.45382239357964e-13	-12.9331769262311
APOE	6.47716733065043e-13	-11.3350242350551
TGM2	2.00763298020276e-11	-9.08344609617999
LTB	4.12409256577302e-10	-7.93889487855185
PROS1	5.05019669643546e-10	-11.3894941643395
TLR6	3.66378973485058e-09	-9.2115923041228
DHH	6.51781504287386e-09	-9.82845985937335
PTN	1.38773501058288e-08	-12.4420968539189
SDC4	3.38481174110293e-08	-12.3865097394603
MFGE8	8.01785541121784e-07	-4.9176517795208
NECTIN2	8.01785541121784e-07	-10.6576688826516
COL1A1	1.02856733089209e-06	-7.31931823725874
COL5A1	1.04074682575387e-06	-10.3563148597998
GDF1	1.19566194671009e-06	-4.73754172975933
HSPG2	1.81860836059974e-06	-9.88878539165365
EPHA5	2.55250477486149e-06	-10.1299298709972
ROR2	2.68926647944338e-06	-3.84450084231794
COL6A2	2.94167486662504e-06	-9.3567029976642
EGFR	2.94167486662504e-06	-2.28660595600028
SPON2	2.94167486662504e-06	-2.59568110674211
VCAN	4.19540860491222e-06	-3.48970852518377
DLL4	4.19540860491222e-06	-8.23157635780941
SERPING1	4.90845107478402e-06	-10.008704269859
IL27RA	2.50958807745131e-05	-8.32776361019137
CDH2	2.98747026891912e-05	-7.55594042048403
COL4A3	3.07469796414594e-05	-10.0104612028334
LRP8	3.16122318130711e-05	-2.7934935537236
COL4A4	3.56425325935595e-05	-2.82020866745299
MIF	4.75348636947746e-05	-9.1276437741678
GPC1	4.94622342913924e-05	-8.17797237626063
COL3A1	5.03899382779905e-05	-4.86239040964847
ITGAV	9.62426037503454e-05	-6.34481277225375
AGRN	0.000122709781606723	-8.01709440898507
VEGFA	0.000177150112982801	-8.6128810754718
ADAM15	0.000177150112982801	-6.65938283066492
PDGFC	0.000239797246250478	-9.5545444359281
MDK	0.000256967969108357	-7.9567804886453
COL4A1	0.000273959673435022	-2.68810040736254
GPC6	0.000420122102957819	-2.45322060836139
TNFRSF1A	0.000440756226485101	-7.26813601141126
LOC534578	0.000542880303560761	-1.81100701151629
PDGFRA	0.00060997004362191	-5.08478953528356
TGFB3	0.000693647556883353	-6.08289935378398
LTF	0.000750542995228625	-7.33596358864902
INSR	0.000791669631064514	-4.90807783785702
CHRM3	0.0010254683493746	-3.37866021920687
NRP1	0.001256752766466	-6.97552518407492
SCARB1	0.00133885368041131	-7.50328664785006
AXL	0.00165854071887997	-5.97784577212295
IL6ST	0.00166935570431297	-2.0900116617505
LRP5	0.00198219160030225	-5.27079956737119
TGS1	0.00205954453611458	-1.37076896807124
CALR	0.00220157956048764	-1.31393541363209
VEGFB	0.00236460158994938	-6.64787223301675
LRP1	0.00236460158994938	-2.00623142808633
PLXNB2	0.00247200336564883	-6.85392685044703
TYRO3	0.00254132996431239	-6.19171312129373
NECTIN1	0.00268140641551392	-5.64459298100141
THBS2	0.00290425283847044	-6.08101936619304
UBC	0.0031821923825082	-1.10586529883496
BAMBI	0.00360273019396387	-1.66032839653726
GNAI2	0.00382347924072888	-3.58655045417657
LDLR	0.00445809974473106	-5.76592267791961
LAMA1	0.00489318434876764	-6.83403931192122
LTBP3	0.00584994256111816	-5.9046970708799
EPHA6	0.00613518586458957	-2.60731972644753
GIPC1	0.00628769099553327	-5.39590399877169
RAMP2	0.00693888161214972	-5.89210740086373
APP	0.00750709532233889	-2.75264984603357
LAMB2	0.00843428828451726	-4.8524259096661
CAP1	0.00923174598201813	-1.05058057422452
FZD1	0.00994793186226189	-5.86110952243636
IGFBP4	0.0104211861466636	-3.75200424717016
SEMA7A	0.0140566014906723	-1.40126926431158
PLXNA1	0.0263951548493891	-4.31405051966082

Differentially expressed ligand and receptors in SFO

gene	adj_p_value	log2FC
ITGB4	4.59949832836107e-14	10.6476282582721
HBEGF	3.74601409724767e-13	6.34970198584512
IL1R1	3.94679531633557e-13	9.61011280030202
CSF1R	1.09721300311561e-11	11.1457593501553
KIT	3.5332750802881e-10	6.39696255301006
DCC	4.29998426071185e-10	6.42627481114993
CDH1	4.63831384120409e-10	4.63669808847857
PSPN	5.05019669643546e-10	4.82655057335601
WNT7A	9.54302723236636e-10	7.66494776317131
VWF	3.68850628963537e-09	8.46182780919972
SDC3	7.43699987373323e-09	5.40485229620385
ACVR1	1.55407386906676e-08	4.10935269300002
BMP6	4.58435310431662e-08	6.1576188542996
MUSK	5.96610849724794e-08	6.43756156077074
FGFR2	6.33856945991457e-08	4.30738320228374
RSPO1	1.02856733089209e-06	9.43703451257352
TNFRSF21	2.27668061155531e-06	3.8030978402831
LAMA2	2.94167486662504e-06	4.96923519198046
ITGA9	6.29527833046029e-06	2.46968531076599
TGFA	1.00840131038637e-05	3.6314858534284
NRG4	2.50958807745131e-05	3.0294510495241
NRG2	2.51628117837006e-05	2.82527904901302
CD36	3.56425325935595e-05	4.41708817472539
COL1A2	5.69836922305675e-05	5.15064867006016
FZD6	9.116900207168e-05	3.77074669439454
SFRP1	0.000122709781606723	4.20998606522853
SDCBP	0.000287587747724359	1.09375199887037
ACTR2	0.000353013732248213	1.59676963805041
TIE1	0.00166935570431297	6.12557307573875
PTCH1	0.00220293676823335	2.24725581642982
SPP1	0.00382347924072888	4.14918072977038
DDR2	0.010369052083157	1.72818354589222
NOTCH4	0.02723554636809	1.22793427008138
TGFBR3	0.029052896481758	1.40282436540443
AMN	0.0377892489800206	3.46710770566183
HSPA8	0.0448463675336202	0.669786642591554

Correlation of LRPs with significant differences between SFGC and SFO expressions

Ligand in SFGC	Receptor in SFO	R	pval	Ligand in SFO	Receptor in SFGC	R	pval
AGRN	MUSK	0.714285714285714	0.0230020525697288	ACTR2	LDLR	0.571428571428571	0.0260955280760047
APP	TNFRSF21	0.428571428571428	0.074642756686281	CD36	TLR6	0.535714285714286	0.116657365969703
DHH	PTCH1	0.607142857142857	0.0243056279181052	CDH1	CDH2	0.464285714285714	0.00901438629886966
INHA	TGFBR3	0.857142857142857	1.81517849244174e-05	COL1A2	ITGAV	0.428571428571428	2.77493259732254e-06
TGFB3	TGFBR3	0.535714285714286	7.6070084947748e-05	HSPA8	LDLR	0.571428571428571	0.0260955280760047
VEGFA	ITGA9	0.464285714285714	0.0633104287388877	SPP1	ITGAV	0.5	2.32000842135626e-06
GIPC1	TGFBR3	0.75	3.48119221460473e-05	TGFA	EGFR	0.607142857142857	1.96229890648114e-06
APOA1	AMN	0.428571428571428	0.0496668476697976	NRG2	EGFR	0.535714285714286	2.44021211272196e-06
COL3A1	DDR2	0.464285714285714	0.0351570749979314				

PSPN	GFRA1
PSPN	SDC3
RSPO1	FZD8
SEMA3B	NRP2
SEMA7A	ITGB1
SERPING1	LRP1
SFRP1	FZD6
SHBG	SLC37A1
SPON2	ITGAM
SPP1	ITGA9
SPP1	ITGAV
SPP1	ITGB1
SPP1	ITGB3
TGFA	EGFR
TGFB1	ACVRL1
TGFB3	ACVRL1
TGFB3	ITGB1
TGFB3	ITGB5
TGFB3	TGFBR3
TGM2	ITGB1
TGM2	SDC4
TGS1	RXRA
THBS2	ITGB1
UBB	FGFR2
UBB	LDLR
UBB	TGFBR1
VCAM1	ITGB1
VCAN	EGFR
VEGFA	ITGA9
VEGFA	ITGB1
VEGFA	NRP2
VEGFC	ITGA9
VWF	ITGB3
WNT5A	FZD1
WNT5A	LRP5
WNT5A	ROR2
WNT7A	FZD5
DLL4	NOTCH2
NECTIN2	NECTIN3
NECTIN1	NECTIN3
UBB	ERBB4
UBC	NOTCH2
UBB	NOTCH2
UBB	MET
FYN	KIT
FYN	EPHB2
VEGFA	AXL
FYN	DCC
FYN	CSF1R
CAP1	ROBO1
NRG2	EGFR
GIPC1	TGFBR3
FYN	PLXNA1
FYN	PLXNA4
NRP1	PLXNA3
TOLLIP	IL1RAP
TGFB3	ITGAV
IL6ST	IL27RA
APOA1	CUBN
APOA1	AMN
MDK	NOTCH2
APOA1	SCARB1
TGFB1	BAMBI
BSG	ITGA6
PROS1	MERTK
SDCBP	EPHB4
MIF	CD74
ANGPT1	TIE1
BMP6	ACVR1
COL3A1	DDR2
PROS1	TYRO3
PTN	PLXNB2
TGFB1	ENG
TGFB3	ENG
TGM2	ADGRG1
VCAN	SELP
VEGFB	TYRO3
WNT5A	ROR1

ADCY9	0.0299538000788548	-1.36778144811909
ITGA3	0.0321423704556785	-4.10925111428901
ITGA6	0.0342410347841196	-1.85232725112093
INHBA	0.0346851050123191	-3.801803892703
BSG	0.0432292696292615	-1.62350588549903

ST1B

**BulkSignalR LRPs**	
Ligand	Receptor
ADAM10	AXL
ADAM15	ITGAV
ADAM15	ITGB1
AGRN	LRP2
APP	CD74
APP	GPC1
ASIP	F11R
COL18A1	GPC1
COL18A1	ITGA3
COL18A1	ITGB1
COL1A2	CD36
COL1A2	ITGA11
COL1A2	ITGAV
COL1A2	ITGB1
COL3A1	DDR1
COL3A1	ITGA2
COL3A1	ITGB1
COL4A1	ITGAV
COL4A1	ITGB1
COL4A3	CD47
COL4A3	ITGA3
COL4A3	ITGB1
COL4A4	CD47
COL4A4	ITGB1
COL6A2	ITGB1
COLQ	MUSK
CUBN	LRP2
DCN	MET
DHH	HHIP
EFNA2	EPHA10
EFNA4	EPHA10
EFNB2	EPHB2
EFNB2	EPHB6
F2	F2RL1
FABP5	RXRA
FGF11	FGFR2
FST	BMPR1B
GDF1	ACVR1B
GDF1	ACVR2A
GDF1	ACVR2B
GDF1	SMAD3
GNAS	ADCY9
GPC6	LRP2
HSPA8	LDLR
IGF2	IGF1R
IGF2	IGF2R
IGFBP4	LRP6
INHA	ACVR2B
IRAK4	TLR6
LAMA1	GPC1
LAMA2	ITGA2
LAMA2	ITGB4
LCN2	LRP2
LIN7C	ABCA1
LIPC	LRP1
LRP1B	PLAUR
LTB	TNFRSF1A
MFGE8	ITGAV
NRG3	ERBB2
PDGFB	ITGAV
PDGFC	PDGFRA

**Differentially expressed ligand and receptors in EAcGC**		
gene	adj_p_value	log2FC
PDGFB	3.27904594661581e-37	-10.4974018306899
INHA	6.49155483806333e-12	-14.9864177610877
APOA1	4.20934765487961e-11	-11.9963101346802
GDF1	1.71683453898506e-10	-4.30473293667433
LTB	2.02024222554819e-10	-7.37934373563518
COL18A1	1.11917754954232e-09	-10.8330827920452
FST	3.97946111695273e-09	-15.0096729264398
VCAM1	1.55098558006028e-08	-4.5586088600403
ANGPT4	1.91961365830558e-08	-8.31125364608164
ITGB8	2.69151810299121e-08	-8.79957385608251
SDC4	4.82642986680495e-08	-11.2761526118615
TGM2	5.81359771081428e-08	-7.91409091187808
TLR6	8.86076907092633e-08	-8.65000631698833
DHH	1.92851439847371e-07	-8.42866248998468
VEGFA	6.28502376180452e-07	-9.42479315660533
LDLR	2.24522133662208e-06	-9.6295252019638
ERBB2	2.57847700228629e-06	-6.84423708469177
DCN	3.01011038568576e-06	-9.43343450271357
COL6A2	4.88792619556269e-06	-8.49403747619328
IGFBP4	4.88792619556269e-06	-5.86142589051491
ADAM15	4.88792619556269e-06	-6.00613139135578
STRA6	9.42689683228762e-06	-11.3038947460011
PLXNB2	1.52971446311633e-05	-8.93693014794683
PROS1	2.08475941121881e-05	-9.0821053212525
PTN	7.84009076531246e-05	-9.30139760394431
FGF11	0.000101852935832871	-7.28442056703427
VIPR2	0.000101852935832871	-6.4598808976023
LCN2	0.000112960897079961	-6.19778296117426
MFGE8	0.000180944245193903	-8.58091119073914
COL4A2	0.000191773972506999	-7.08880441678992
GPC1	0.000301675192072161	-7.23630774633182
DDR1	0.000301675192072161	-7.46381516406972
LAMA1	0.000420948479042867	-8.2077625025635
SCARB1	0.00045019949553828	-7.98536160389686
VCAN	0.000489126967074393	-7.2345336570033
AGRN	0.000640147459167327	-6.70824035740909
COL3A1	0.000960371275148299	-2.92936165926864
TYRO3	0.00211995207783599	-6.8057484473512
RXRA	0.0031841400159845	-5.90576164270246
COL4A3	0.00435103117582806	-7.22820439452085
EPHB4	0.0055187128362915	-4.80364932554933
GRIN2D	0.00735396767565424	-4.07358225936476
ASIP	0.00775543779387854	-4.83983861963133
PDGFC	0.00987242649225273	-5.05511336039417
VEGFC	0.0105168086364549	-4.89714548962484
THBS2	0.0117024253773981	-4.90407299835755
ITGAV	0.0127964959577979	-5.37532294254651
F2	0.014183404828699	-4.67433431886643
PDGFRA	0.0152025641591091	-4.44644696890477
ITGAM	0.0220515701850153	-3.16847987302096
SPON2	0.0257026610089764	-1.77127321088431
UBA52	0.0326843651532628	-3.17044723389578
COL4A1	0.0332564601401405	-4.22393378278016
CUBN	0.0425996082449988	-3.66552792161204
LRP1	0.0425996082449988	-4.24581365507564
PLAT	0.0457969592870023	-3.59170200356863

**Differentially expressed ligand and receptors in EAO**		
gene	adj_p_value	log2FC
BMPR1B	1.40643475934012e-07	3.24070356641171
F2RL1	1.92434837873884e-05	7.18817626600854
LIPC	3.76437661873918e-05	8.45402045661998
AMN	8.42978458630691e-05	6.20715795126608
TGFA	0.000113526305494861	6.95936257336633
DCC	0.000169111425338961	6.62530616773524
ABCA1	0.000472368882866797	7.45521183056219
TIE1	0.000637156315918252	7.14877180575452
ITGA11	0.000848077141987569	5.89086763424696
CD36	0.000941324281253835	5.1816919799712
ITGB4	0.00102630329936439	7.98494339310314
EPHA10	0.00119821678922595	6.8949426576658
MUSK	0.00142570053229751	7.24551878793832
RSPO1	0.00430136591294231	6.2610276788208
EZR	0.00457328575419244	5.49297278906059
WNT7A	0.00935513981111512	5.82138617811389
HHIP	0.0120267447460557	5.29003398358094
FABP5	0.0193830870566216	3.81296170116994

**Correlation of LRPs with significant differences between EAcGC and EAO expressions**							
Ligand in EAcGC	Receptor in EAO	R	pval	Ligand in EAO	Receptor in EAcGC	R	pval
DHH	HHIP	0.8	0.000217546426647825	FABP5	RXRA	0.6	1.84471144226059e-07
F2	F2RL1	0.4	0.0437748023027294	LIPC	LRP1	0.8	0.0107849071046382
FST	BMPR1B	0.8	0.00160868492901005	AMN	CUBN	0.4	0.00497015151295385
THBS2	CD36	0.8	0.000209862858198558				
CUBN	AMN	0.4	0.00552277648955204				
APOA1	ABCA1	1	0				
ANGPT4	TIE1	0.4	0.166070575663678				

PLAT	ITGAM
PROS1	AXL
PTDSS1	SCARB1
PTN	PTPRS
PTN	SDC3
RPS27A	ERBB2
RPS27A	FGFR2
RSPO1	FZD8
RSPO1	LGR4
SEMA3A	PLXNA2
SEMA3B	NRP2
SEMA7A	ITGB1
SPON2	ITGAM
SPP1	ITGB3
TF	LDLR
TGFA	EGFR
TGFB1	ACVRL1
TGFB3	ACVRL1
TGFB3	ITGB1
TGFB3	TGFBR3
TGM2	ITGA9
THBS1	SDC4
THBS2	CD36
THBS2	CD47
THBS2	NOTCH4
TLN1	ITGB3
UBA52	EGFR
UBA52	FGFR2
UBA52	LRP2
VCAN	EGFR
VEGFA	EPHB2
VEGFA	ITGA9
VEGFC	VIPR2
WNT11	FZD7
WNT5A	FZD8
WNT5A	RYK
WNT7A	FZD5
VCAM1	MSN
WNT5A	FZD3
RPS27A	ERBB4
UBA52	MET
FYN	EPHB4
FYN	DCC
COL4A2	ITGAV
LTBP1	ITGB8
TGFB3	ITGAV
DLG3	GRIN2D
AMN	CUBN
CUBN	AMN
TNFSF12	TRAF3
BSG	ITGB1
BSG	ITGA6
SDCBP	EPHB4
EZR	DCC
APOA1	ABCA1
ANGPT4	TIE1
PROS1	TYRO3
PTN	PLXNB2
RBP4	STRA6
RTN4	TNFRSF19
VCAN	SELP
