# Supplementary material for: Dynamic Remodeling of Oocyte‐Granulosa Cell Communication During Bovine Folliculogenesis Revealed by Transcriptomic Meta‐Analyses
Source: FASEB J. 2026 Sep 11;40(18):e72289. doi: 10.1096/fj.202603316RR (PMC13564240; doi:10.1096/fj.202603316RR)
Supplement: Supplementary file 1 [file FSB2-40-e72289-s001.zip › 202603316RR-sup-0005-SI_Figure-S01.pdf]

Additional File 2

Fig. S1A

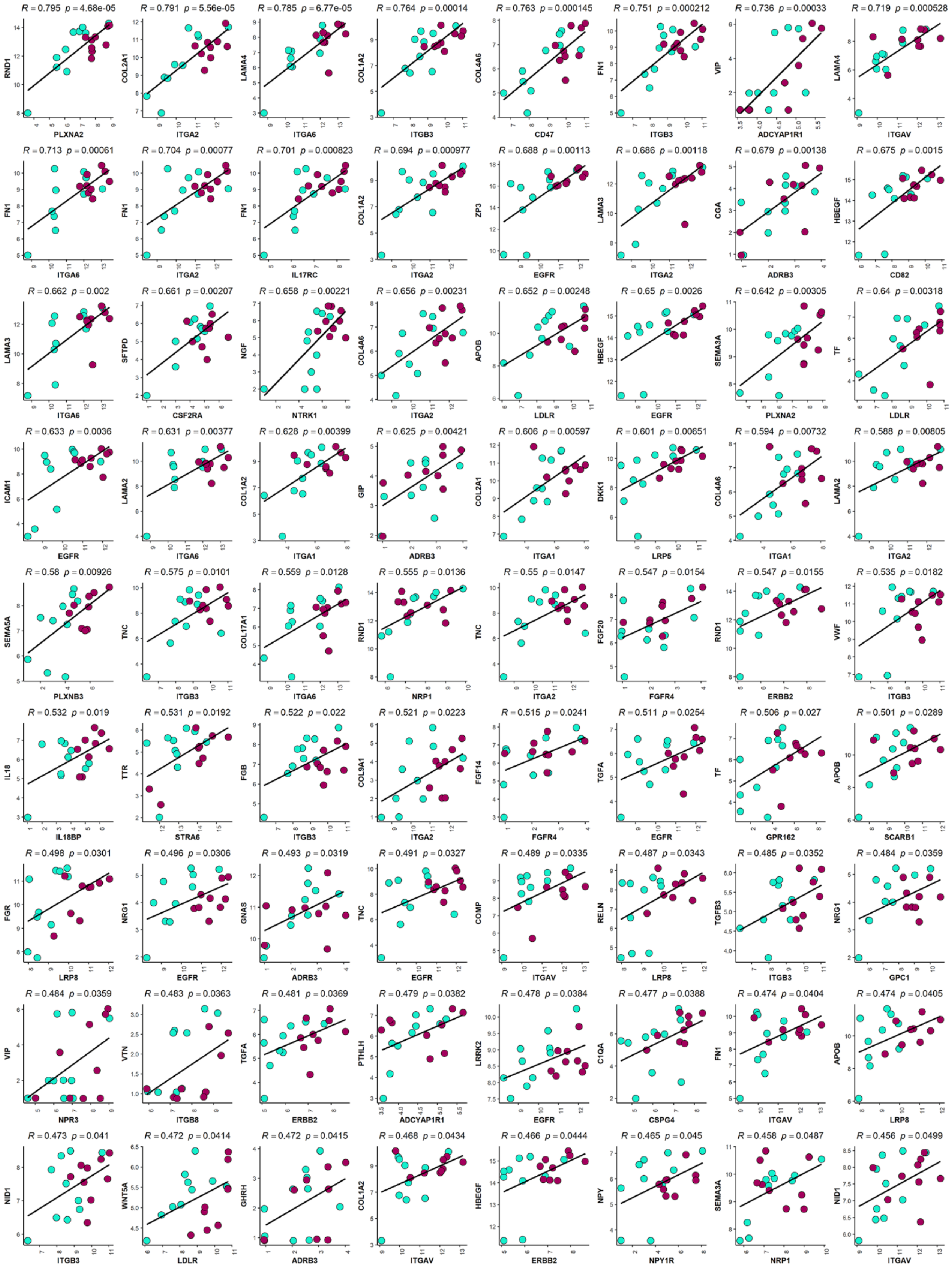

Additional File 2

Fig. S1B

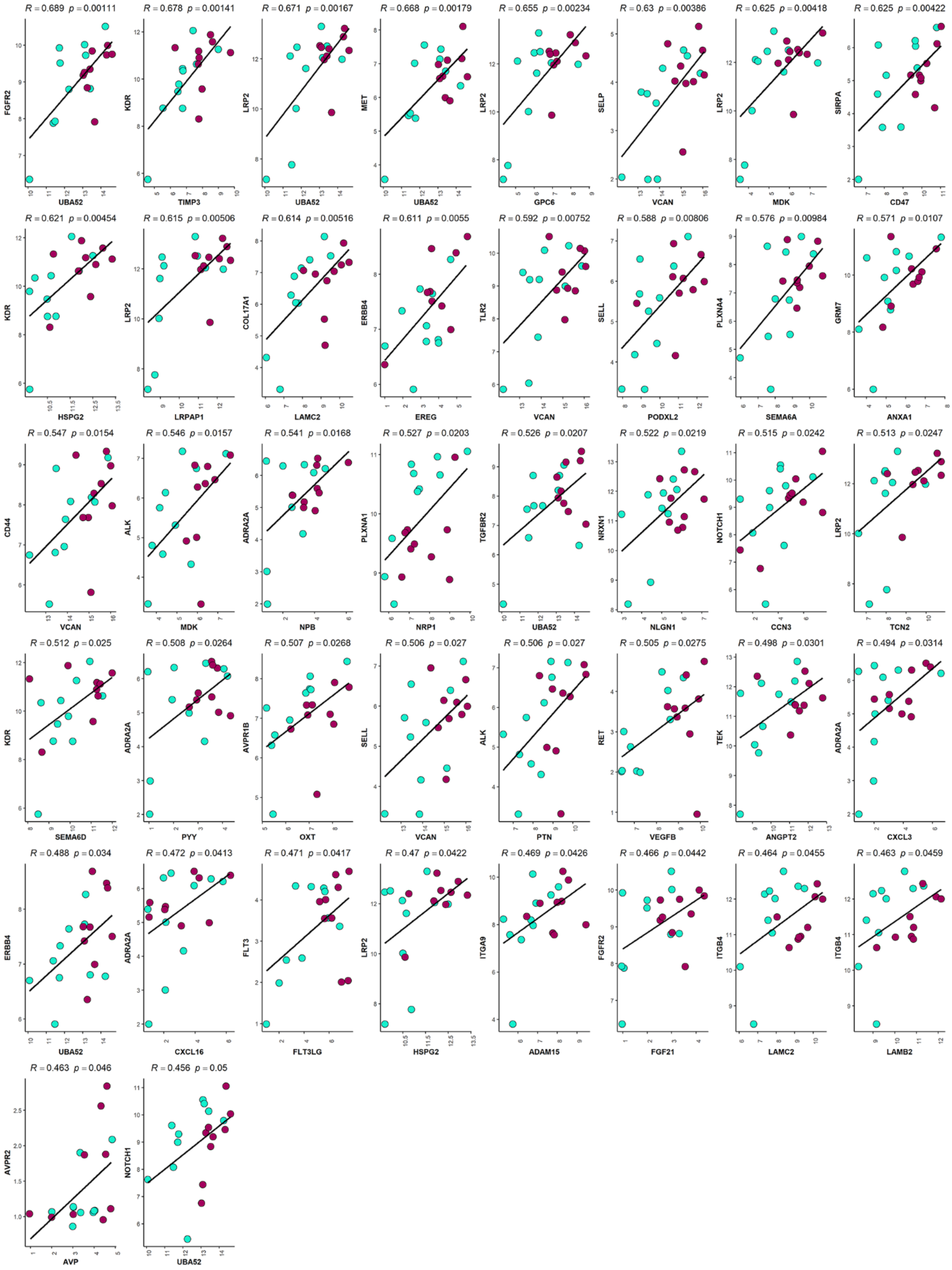

Additional File 2. Ligand receptor positive correlations. **Fig. S1A** MAcGC receptors positively correlated with MAO ligands. **Fig. S1B** MAcGC ligands positively correlated with MAO receptors. The correlation graphs display all positive Pearson correlations; the correlation coefficient (R) and corresponding *p* are indicated at the top of each graph.
