# Supplementary material for: Dynamic Remodeling of Oocyte‐Granulosa Cell Communication During Bovine Folliculogenesis Revealed by Transcriptomic Meta‐Analyses
Source: FASEB J. 2026 Sep 11;40(18):e72289. doi: 10.1096/fj.202603316RR (PMC13564240; doi:10.1096/fj.202603316RR)
Supplement: Supplementary file 1 [file FSB2-40-e72289-s001.zip › 202603316RR-sup-0006-SI_Figure-S02.pdf]

Additional File 3

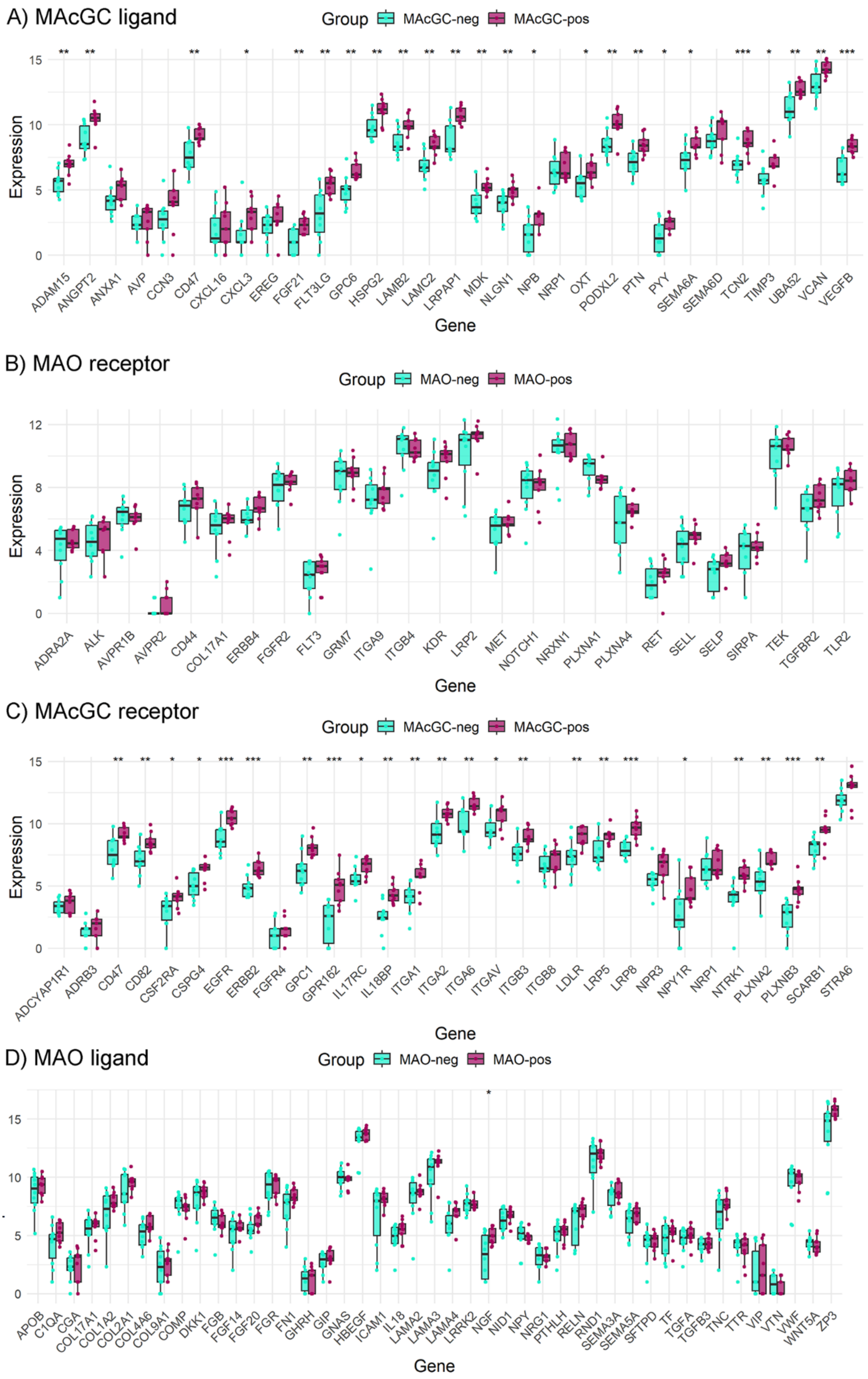

Additional File 3. Differential gene expression of ligand and receptors based on BCB staining. Box plots show the differential expression of LRPs according to BCB staining.

Additional File 3

Expression differences are reported for **A** MAcGC ligands and **B** MAO receptors, **C** MAcGC receptors and **D** MAO ligands. Turquoise indicates expression levels in MAO-neg and MAcGC-neg, while purple indicates those derived from MAO-pos and MAcGC-pos. Statistical significance was assessed using ANOVA. Asterisks denote levels of statistical significance (* $p<0.05$; ** $p<0.01$; *** $p<0.001$).
